# A versatile microfluidic tool for the 3D culture of HepaRG cells seeded at various stages of differentiation

**DOI:** 10.1038/s41598-021-92011-7

**Published:** 2021-07-07

**Authors:** Manon Boul, Nassima Benzoubir, Antonietta Messina, Rasta Ghasemi, Ismail Ben Mosbah, Jean-Charles Duclos-Vallée, Anne Dubart-Kupperschmitt, Bruno Le Pioufle

**Affiliations:** 1 Université Paris Saclay, ENS Paris Saclay, CNRS SATIE, 4 avenue des Sciences, 91190 Gif-sur-Yvette, France; 2 UMR_S 1193 INSERM/Université Paris-Saclay, 94800 Villejuif, France; 3 Université Paris Saclay, ENS Paris Saclay, CNRS LUMIN, 91190 Gif-sur-Yvette, France; 4 FHU Hépatinov, Centre Hépato-Biliaire, Hôpital Paul Brousse, 94800 Villejuif, France; 5 Université Paris Saclay, Institut D’Alembert, ENS Paris Saclay, CNRS, 91190 Gif-sur-Yvette, France; 6 Biopredic International, Parc d’Affaires La Bretêche, 35760 Saint-Grégoire, France; 7 APHP, Centre Hépato-Biliaire, Hôpital Paul Brousse, 94800 Villejuif, France

**Keywords:** Lab-on-a-chip, Tissue engineering, Liver

## Abstract

The development of livers-on-a-chip aims to provide pharmaceutical companies with reliable systems to perform drug screening and toxicological studies. To that end, microfluidic systems are engineered to mimic the functions and architecture of this organ. In this context we have designed a device that reproduces series of liver microarchitectures, each permitting the 3D culture of hepatocytes by confining them to a chamber that is separated from the medium conveying channel by very thin slits. We modified the structure to ensure its compatibility with the culture of hepatocytes from different sources. Our device was adapted to the migratory and adhesion properties of the human HepaRG cell line at various stages of differentiation. Using this device, it was possible to keep the cells alive for more than 14 days, during which they achieved a 3D organisation and acquired or maintained their differentiation into hepatocytes. Albumin secretion as well as functional bile canaliculi were confirmed on the liver-on-a-chip. Finally, an acetaminophen toxicological assay was performed. With its multiple micro-chambers for hepatocyte culture, this microfluidic device architecture offers a promising opportunity to provide new tools for drug screening applications.

## Introduction

The use of animal models and two dimensional (2D) cell cultures usually fail to reproduce in vivo drug metabolism and liver toxicity in humans^1^. Indeed, the correspondence between animal and human models regarding hepatic drug toxicity only reaches 30 to 50%^1,2^. Furthermore, primary hepatocytes cultured under 2D conditions rapidly lose their functions and cannot offer a long term assessment of medicinal effects^3^. Lastly, hepatotoxicity is considered as one of the principal reasons for the withdrawal of FDA-approved drugs from the market^4^. The pharmaceutical industry therefore requires access to improved predictive models.

Culturing hepatocytes under flux and/or three dimensional (3D) conditions has been shown to improve their viability and functions^5^. For this reason, perfused hepatocyte culture systems are currently seen as promising candidates to reflect liver functionality in vitro^6^. Because of their small size, liver-on-a-chip devices offer a convenient tool to culture hepatic cells under a flow and produce an adequate supply of high-throughput data for drug development^7,8^. Using such systems, cells are generally cultured as either 2D monolayers, spheroids or aggregates within an oversized chamber^9–16^. Several research groups have taken advantage of microfluidic techniques to reproduce the microarchitecture of the liver. In particular, a hepatocyte cord was mimicked on a chip^17–19^; the dimensions of the cell culture chamber and its proximity to a vascular channel were more similar to the in vivo situation than other systems. However, a small number of primary hepatocytes or HepG2 cells were seeded and only their 2D organisation could be achieved using these devices.

The development of a model that can predict liver drug metabolism and be used for toxicity studies requires considerable accuracy and reproducibility. This is reliant on the quality of the hepatocytes used in the systems described above, but how to obtain an unlimited supply of high quality hepatocytes still needs to be clarified^6^. For this reason, it is necessary to develop a versatile device compatible with human hepatocytes from different sources (including primary cells, liver tumour-derived cell lines, and cells derived from induced pluripotent stem cells (iPSCs)) in order to compare their responses to drugs^1,6^.

To better reflect the functions of the liver, we propose in this paper to combine the on-chip mimicking of hepatic-cord microarchitecture with the 3D structuration of hepatocytes, playing on the geometry of the culture chambers. We also propose to multiplex this structure in order to simultaneously culture tens of organised tissues and measure hepatocyte albumin secretion. Such combined features have never been demonstrated previously. For the first time, this hepatocyte-cord-like microfluidic chip was used to culture the HepaRG cell line. Of all the human hepatic cell lines available, differentiated HepaRG cells are considered to be the most similar to human primary hepatocytes in terms of their functions and level of metabolic activity^6,20^. When seeded at low density, HepaRG cells are proliferative hepatoblast-like cells, but once they reach confluency, they undergo spontaneous differentiation into hepatocyte-like cells. This differentiation can be enhanced by adding dimethyl sulfoxide (DMSO) to the culture medium. Both hepatoblast- and hepatocyte-like HepaRG cells are already widely used for liver bioreactor applications^21,22^ and to evaluate drug toxicity^23–26^, respectively. The properties of hepatoblasts and hepatocytes from the HepaRG cell line were evidenced to differ in terms of their migration and adherence: this feature prompted us to adapt the structure and packaging of the chip accordingly, and the culture of these cells under flux for several weeks was then achieved. The viability and spontaneous differentiation of cells into hepatocytes were assessed in the microfluidic device, as was their 3D organisation. Finally, the liver-on-a-chip sensitivity to an acetaminophen exposure was investigated.

## Results

### Design of the device

**Figure 1:**
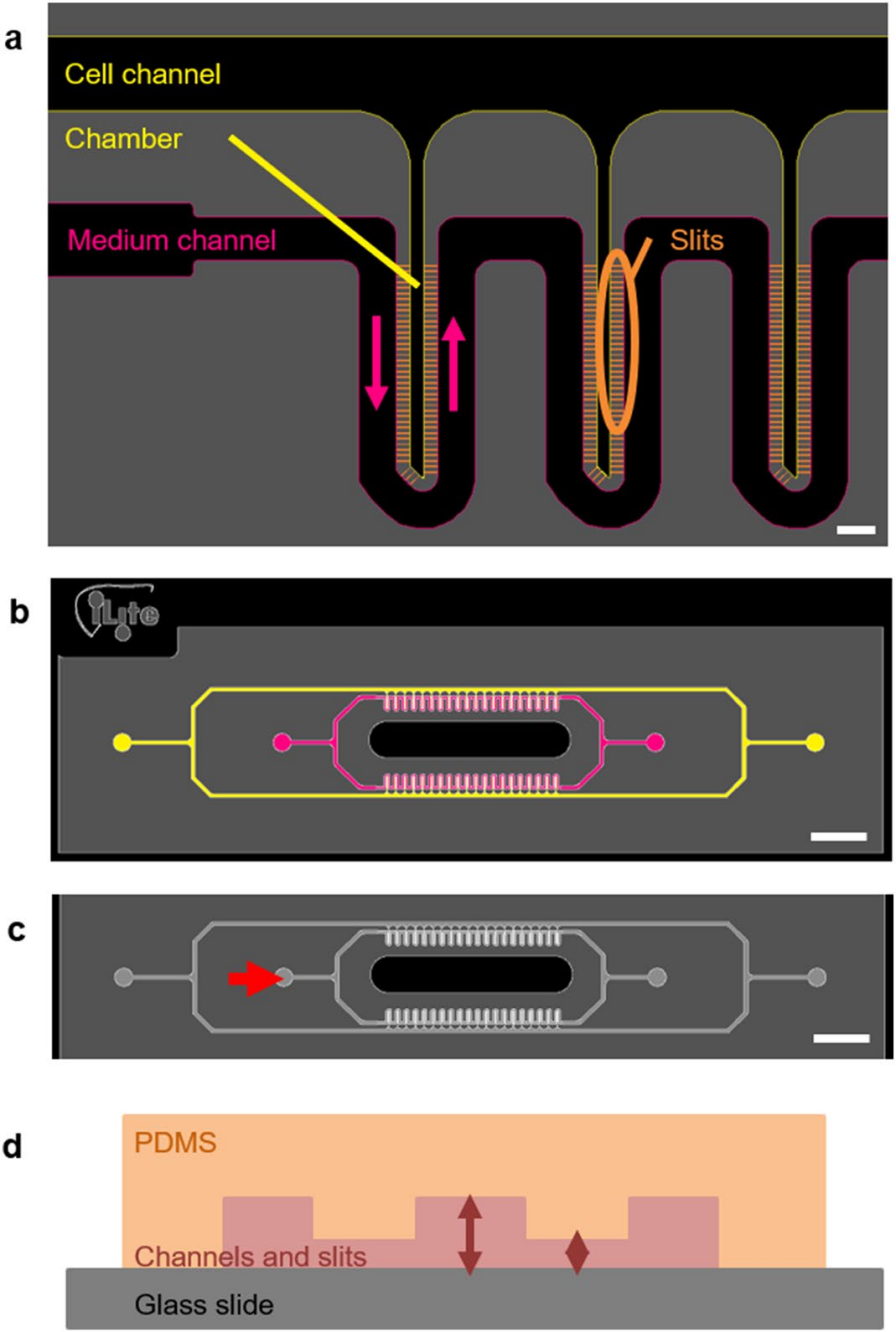
Design of the microfluidic device. (**a**) Detailed view on three chambers. The cell channel and hepatocyte chambers (yellow) communicate with the medium channel (pink) via an array of slits (orange). The direction of medium flow is represented by pink arrows. Scale bar = 100 µm. (**b**) Top-down view of the design which comprises two fluidic circuits: in pink the medium channel and in yellow the cell channel. Scale bar = 3 mm. (**c**) Fluidisation of the chip: the flow enters from one inlet of the medium channel (red arrow) and leaves via three outlets. Scale bar = 3 mm. (**d**) Side-on diagram of the device: a polydimethylsiloxane (PDMS) chip (orange) is sealed to a glass slide (grey), thus creating microfluidic channels (pink). Depending on the different versions of the device, the slits are 2 or 5 µm high and the other channels are 25 or 40 µm high.

Our device was designed to reproduce the liver microarchitecture and was based on proposals previously described by Lee et al*.*^17^ and Nakao et al*.*^18^: a cell culture chamber (38 µm in width) is surrounded by a medium conveying channel and both being linked by arrays of slits (Fig. 1a). These elements mimic hepatic cords, liver sinusoids, and the space of Disse, respectively. The slits enable oxygen and nutrients to diffuse to the cells while protecting them from high shear stress. Under our design, the height of the central chamber is optimised in order to promote the 3D organisation of hepatocytes. To maximise the number of hepatic cords on a single microfluidic device, this unit structure is arranged as two parallel series of 20 hepatocyte culture chambers. Our device comprises two fluidic circuits that are interconnected via the slits: a medium flowing channel and a cell loading channel, to enable the seeding of multiple chambers (Fig. 1b). These fluidic circuits are filled and controlled from the four outlets placed at the extremities of the microfluidic chip. Our initial device was designed with channel and slit heights of 25 and 5 µm (Fig. 1d), respectively, so is thus referred to as the “25–5 µm device”. In this device, the slits were 5 µm high, 3.5 µm wide and 37 µm long.

### Device optimisation to prevent cell migration through the slits and promote their 3D organisation

#### Migration of HepaRG hepatoblasts on chip depending on slit dimensions

We first of all loaded HepaRG cells at a proliferative stage onto the device: we expected them to adhere to the glass and PDMS, proliferate and then completely fill the chambers. Because these cells have a diameter of around 17 µm^20^, they were expected to organise themselves in 3D structures with stacks of two cells, and once they reached a high density to spontaneously differentiate into hepatocytes.

**Figure 2:**
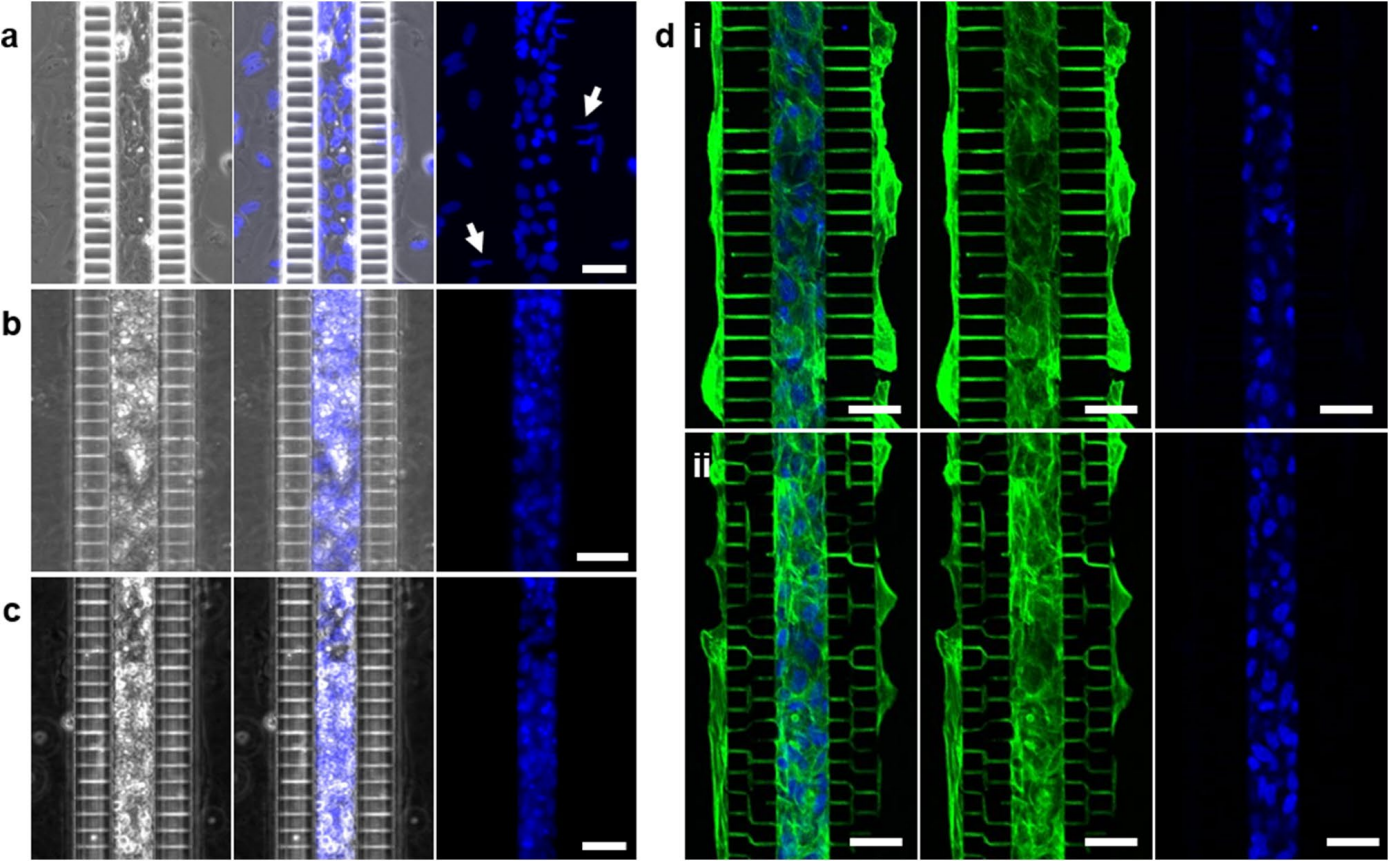
Evidence for the migration of proliferative HepaRG cells out of culture chambers determined by phase contrast imaging and fluorescent staining (blue: DAPI, green: phalloidin, staining cell nuclei and F-actin, respectively). Proliferative HepaRG cells loaded and observed after 2 days under static conditions, in different devices: (**a**) on a 25–5 µm device, the migration of cell nuclei through the slits was observed (white arrows); (**b**) on a 25–2 µm device; (**c**) on a 40–2 µm device. (**d**) Confocal microscope images of a chamber on the 40–2 µm device in the same conditions: (i) with straight slits or (ii) with discontinuous slits. Scale bars = 40 µm.

After loading the cells onto the 25–5 μm device and culturing them under static conditions, they were found to migrate out of the chambers through the slits (Fig. 2a). The absence of HepaRG cells from the medium channel immediately after loading and the presence of nuclei within the slits after several hours of culture provided evidence of cell migration. Once inside the medium channel, the cells started to adhere, proliferate and fill the channel, which was not our objective. Such observation differed from previous studies with HepG2 cells^19^, which led us to consider that this device was not suited to culturing the cells we were targeting. The design was modified consequently.

To prevent cell migration, the height of slits was reduced to 2 µm, as was their width. A device with 25 µm height chambers was fabricated and referred to as “25–2 µm device”. Under the same static conditions as used for the 25–5 µm device, few nuclei were found outside the chambers or in the slits (Fig. 2b). However, cytoplasmic extensions were seen to elongate through the slits into the medium channel, as demonstrated by phalloidin staining (Supplementary Fig. S1).

To prevent these cytoplasmic extensions, a different design of slits was tested. Of the two series of 20 chambers in the device, one consisted of straight slits (as previously) while the other was made of discontinuous slits including 90° angles (Supplementary Fig. S2). Moreover, to favour the stacking of cells inside chambers, the channel height was increased to 40 µm. A new system, referred to as the “40–2 µm device”, was thus produced. The cell loading protocol led to a homogeneous filling of the chambers with cells (Supplementary Table S1). Surprisingly, when compared to straight slits, discontinuous ones at 90° angles did not reduce these cytoplasmic extensions (Fig. 2c,d).

Finally, decreasing slits dimensions from 5 × 3.5 μm^2^ to 2 × 2 μm^2^ efficiently hindered the migration of proliferative HepaRG cells through them. However, their cytoplasmic extensions could not be prevented, even with a different design of slits.

#### 3D cell organisation in different chamber heights

**Figure 3:**
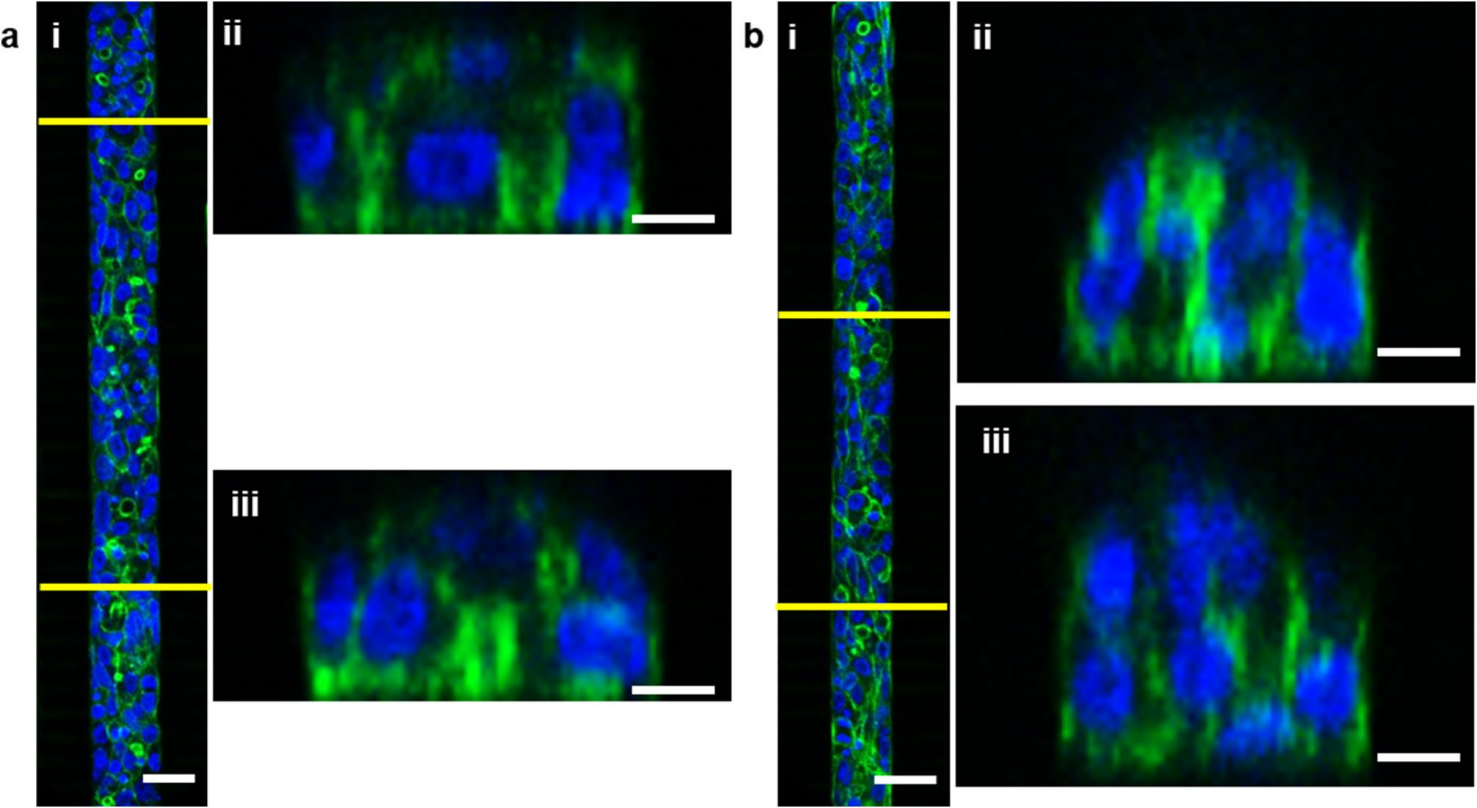
Comparison of cell 3D organisation within two different heights of chambers. Proliferative HepaRG were loaded and observed after 2 days in static conditions by fluorescent staining (blue: DAPI, green: phalloidin) with a confocal microscope. (**a**) Observations on a 25–2 µm device: (i) top-down view, with cross-sections taken at the positions indicated by yellow lines, scale bar = 40 µm; (ii) and (iii) cross-sectional views along the chamber, scale bars = 10 µm. (**b**) Observations on a 40–2 µm device: (i) top-down view, with cross-sections taken at the positions indicated by yellow lines, scale bar = 40 µm; (ii) and (iii) cross-sectional views along the chamber, scale bars = 10 µm.

We wanted to induce the 3D organisation of cells inside the chambers, as this would correspond to more physiological culture conditions. Chambers of 25 or 40 µm in heights were compared. In both the 25–2 and 40–2 µm devices, the cells did aggregate and stack (Fig. 3). However, stacks of only 2 cells were formed inside the 25–2 µm devices compared to stacks of 3 cells inside the 40–2 µm one. Because the latter design was promoting more cell–cell contacts, it was used for the remainder of the study. The percentage of the chamber surface filled with 3D structures was estimated in the 40–2 µm device: on average, 70 ± 21% of a chamber contained cell aggregates thicker than 25 µm.

### Cell culture and differentiation on the chip

#### Theoretical determination of suitable long-term culture conditions

Our device had been initially tested under static condition. However, establishing a flow in the medium channel was mandatory for the long-term culture of cells (Fig. 1c), as evidenced by previous studies^17–19,27^. A flow rate range suitable for the culture of hepatocytes was therefore determined by simulations in the 40–2 µm device. To calculate the maximal flow rate value, the mapping of fluid velocity and shear stress inside the chambers was analysed and is detailed in the Supplementary Information (Supplementary Fig. S3 & S4). In the knowledge that 0.5 Pa is the maximum acceptable shear stress for hepatocytes^28^, the maximum flow that could be imposed was 9.5 µl/min. Moreover, to determine the minimal value of the flux that would keep the cells alive, we estimated the level of their oxygen supply, which was found to arise from its diffusion through polydimethylsiloxane (PDMS) (Supplementary Fig. S5 & S6). Even at a low flow rate, therefore, the cells would receive sufficient oxygen. Ultimately, it was decided that a flow rate lower than 9.5 µl/min was appropriate for the culture of hepatocytes on this device.

#### Desensitising the device to pressure disturbances to culture differentiated HepaRG cells

Compared to proliferative HepaRG cells, HepaRG hepatocytes adhered less to the substrate than to each other^29^. We therefore expected them to be more prone to forming aggregates in the chambers and less likely to invade the slits. However, once these cells were loaded into the 40–2 µm device, the pressure disturbances induced when connecting the tubes to establish the flow drove the cells out of the chambers. Hydrostatic pressure variations induced by modifying the respective heights of the syringe and the device produced similar effects. Increasing the delay prior to fluidisation (from 4 to 24 h), which might have improved cell adhesion to the substrate, was ineffective in preventing this phenomenon. To reduce the flux generated by these pressure disturbances, we added an external flow resistance to our device, which consisted in a thin capillary tube and enabled cells to stay within the chambers and to be cultured for up to 21 days (Supplementary Fig. S7 & S8). Finally, HepaRG cells loaded as both proliferative and differentiated could be successfully loaded and cultured on chip for extensive periods of time.

#### Cell viability and differentiation into hepatocytes

**Figure 4:**
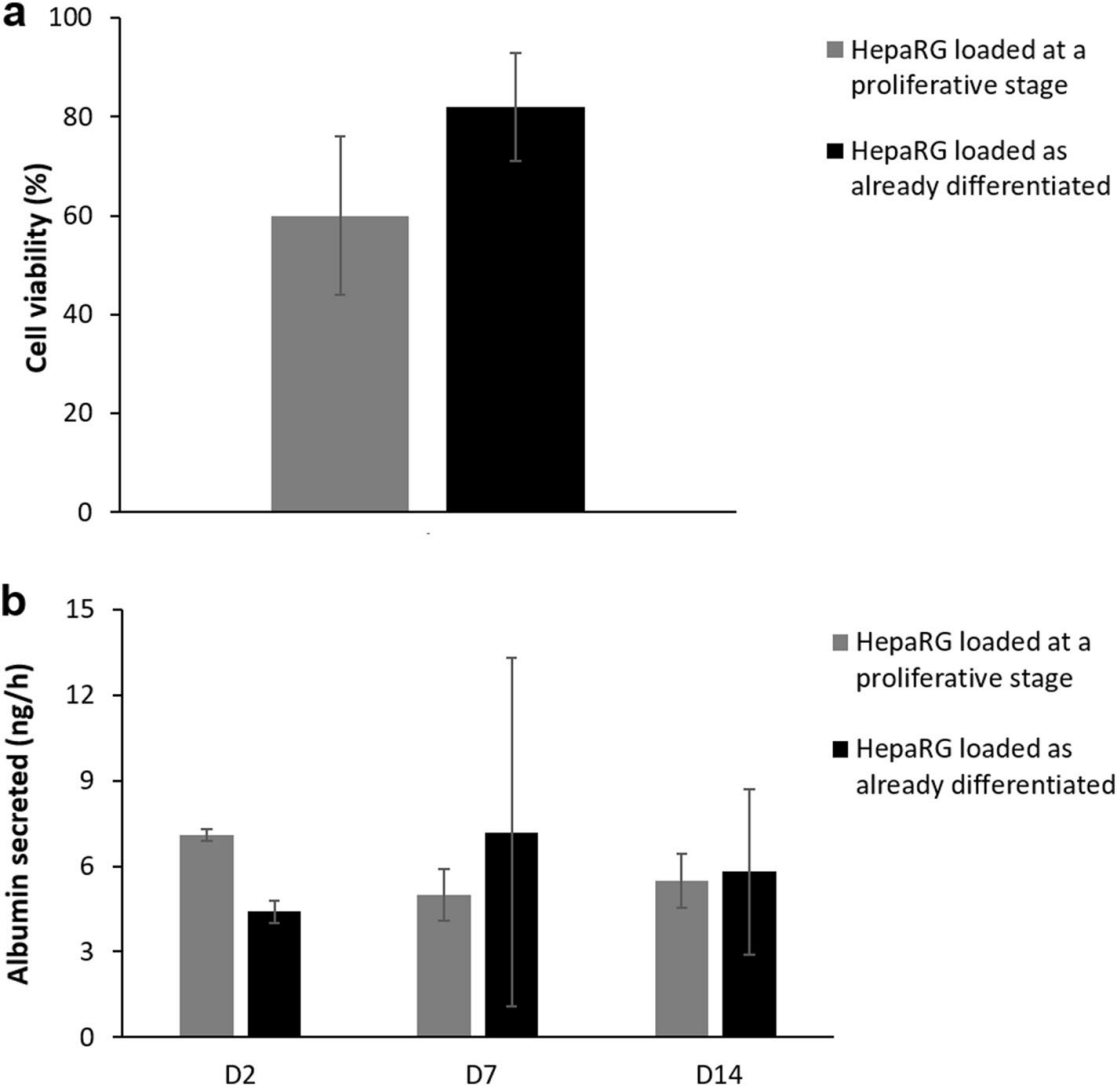
Cell viability and secretion of albumin on chip depending on the differentiation stage at which they were loaded (grey for proliferative HepaRG cells and black for already differentiated cells). (**a**) Cell viability after 15 days of culture on chip (n = 3 chips per condition). (**b**) Secretion of albumin per chip after 2, 7, or 14 days of culture (n = 2 at day 2; n ≥ 3 at day 7 and 14).

The viability of HepaRG cells that had been loaded as either proliferative or differentiated cells was assessed after 15 days of culture on chip: they exhibited 60 ± 16% and 82 ± 11% viability, respectively (Fig. 4a). An example of the assay is provided in Supplementary Fig. S9. Although the viability was lower in the case of HepaRG cells loaded at a proliferative stage, as evidenced by the significant number of apoptotic nuclei, the mean number of live cells inside the chambers was higher than for already differentiated cells (69 ± 26 compared to 52 ± 23, respectively).

The albumin produced by hepatocytes in the chip was quantified at three different time points (day 2, 7 and 14 of culture) for devices loaded with either proliferative or differentiated cells. In both cases, the albumin levels were constant from day 2 to the day 14 of culture (Fig. 4b). No significant differences were evidenced depending on the cell differentiation status at the time of loading.

**Figure 5:**
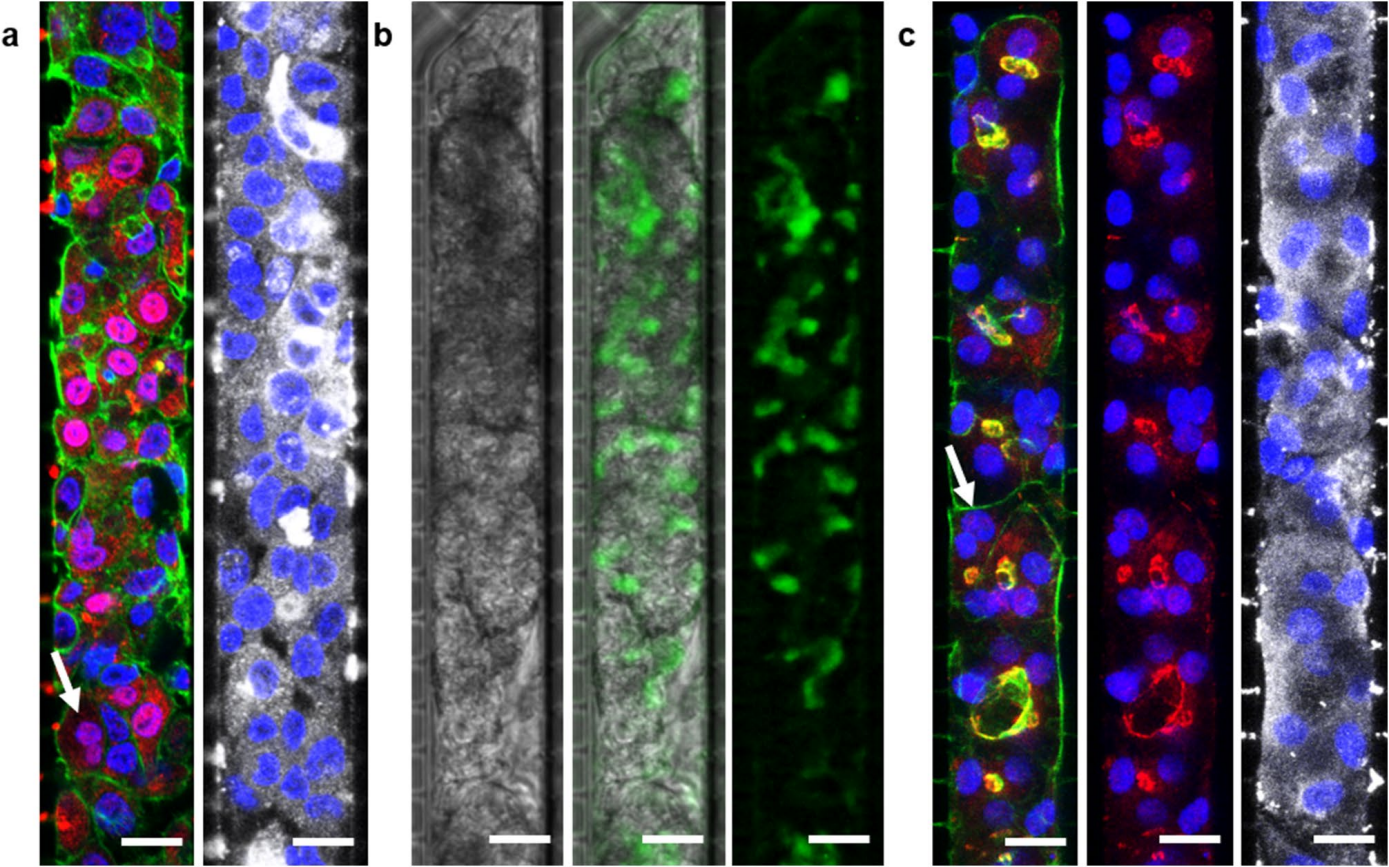
Immunofluorescence or DCFA staining of HepaRG cells cultured on the 40–2 µm device, revealed under epifluorescent or confocal microscopy. Top-down views of the chamber with respect to proliferative HepaRG cells loaded and cultured for 14 days (**a**) fixed and stained for nuclei (blue), F-actin (green), HNF4α (red) and albumin (grey) or (**b**) at 50 nl/min, stained for DCFA (green) and examined under phase contrast imaging. (**c**) HepaRG hepatocytes loaded and cultured for 21 days fixed and stained for nuclei (blue), F-actin (green), ZO-1 (red), and albumin (grey). Some binucleated cells can also be seen (white arrows). Scale bars = 20 µm.

Immunofluorescence assays revealed that after 14 days of culture on the chip, HepaRG cells that had been loaded as hepatoblasts were producing albumin and some of them were positive for HNF4α labelling, particularly in areas of cell 3D aggregation (Fig. 5a). HNF4α is a transcription factor that is expressed early during hepatoblast differentiation into hepatocytes and regulates albumin gene expression. The presence of these two markers indicated that proliferative HepaRG cells were differentiating spontaneously towards hepatocytes on the chip. To visualise the formation and functionality of bile canaliculi, 5(6)-carboxy-2’,7’-dichlorofluorescein (DCFA) was used. This molecule is metabolized and selectively excreted into bile canaliculi by MPR2 transporters. HepaRG cells loaded at a proliferative stage were exhibiting functional bile canaliculi after 14 days of culture on chip (Fig. 5b).

Concerning already differentiated HepaRG cells, after 21 days of culture on the chip, these cells were still able to produce albumin. In addition, some cells exhibited ZO-1 labelling, indicative of the presence of tight junctions. Its co-localisation with F-actin (phalloidin staining) at some points suggested strong interactions and the polarization of cells (Fig. 5c), as expected for hepatocytes. These cells were maintaining their differentiation and function after a long period of 21 days on the device. In both cases, some binucleated cells were visible, which was also an indication of their differentiation into hepatocytes.

#### Acetaminophen toxicological assay

We chronically exposed differentiated HepaRG cells seeded in our device to acetaminophen (APAP) and used a 2D culture of the same cell-type as control. We first established that on HepaRG cultured in 2D for 28 days in the differentiation medium, a 24 h treatment with 20 mM APAP caused the death of 50% of the cells. Through phase contrast microscopy and propidium iodide staining, only the cells differentiated into hepatocytes and forming the refringent clusters were evidenced to be affected by APAP treatment and finally died (Supplementary Fig. S10). It is known that, in 2D, differentiated HepaRG cell culture was composed of 50% of hepatocytes and 50% of other epithelial cells^26^. We therefore assumed that 20 mM APAP was nearly a 100% lethal dose for HepaRG differentiated into hepatocytes. For these reasons and because HepaRG cells cultured in 3D or onto microfluidic chip have been described to be more sensitive to APAP than conventional static 2D cultures^23,38^, we decided to decrease APAP concentration in our on-chip experiments. After 14 days of culture, chips loaded with differentiated HepaRG cells were exposed to a low but constant 2 mM concentration of APAP for 6 days, mimicking a chronic exposure to this drug.

**Figure 6:**
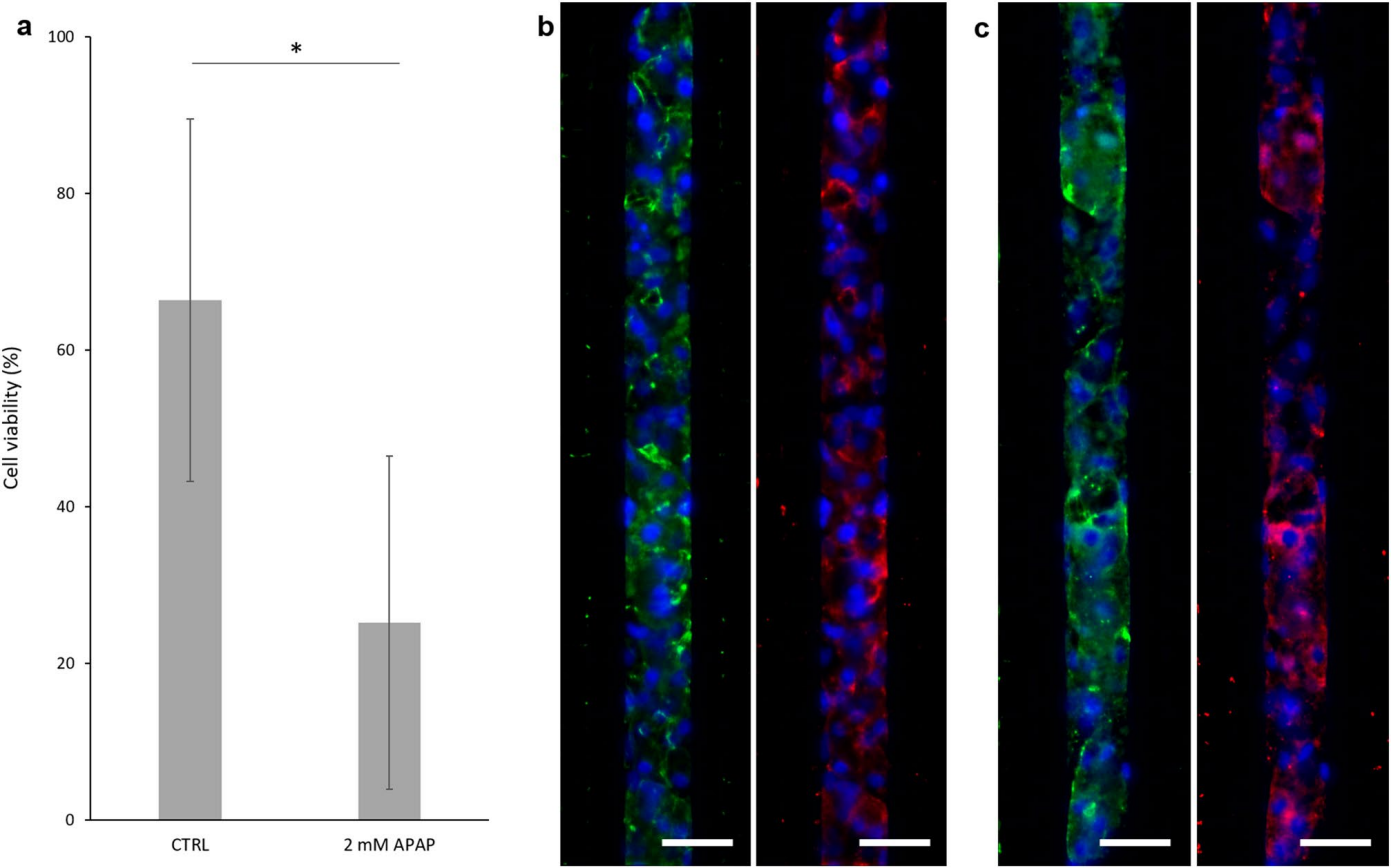
Chip response to acetaminophen (APAP) exposure. Differentiated HepaRG were loaded and culture on chip for 14 days, then exposed to 2 mM APAP for 6 days. Their viability was assessed by a Live and Dead test, they were then fixed and stained for nuclei (blue), ZO-1 (green) and MDR3 (red). (**a**) Cell viability in control and treated chips (CTRL n = 3 chips, 2 mM APAP n = 2 chips). Immunofluorescent images of (**b**) a control chip, (**c**) a chip treated with APAP. Scale bars = 40 µm. *p < 0.01.

Cell viability was significantly reduced after 6 days of treatment (Fig. 6a). Indeed, cell aggregated within the chambers were evidenced to dissociate and even detach, as some of them were expelled from the chambers (Supplementary Fig. S11). Experiments carried out in 2D highlighted that hepatocytes derived from HepaRG and treated with APAP eventually lost their tight-junctions (Supplementary Fig. S12). This resulted in the loss of their polarity and bile canaliculi, ultimately leading to their death and detachment. From immunostaining analysis, we could evidence that the same phenomenon was occurring in our device: in the APAP-treated chambers tight-junction (ZO-1 marker) and bile canaliculi transporter (MDR3 marker) labelling appeared faint and less defined as compared to the non-treated chips (Fig. 6b,c and Supplementary Fig. S13). Thus, HepaRG hepatocytes in our devices were demonstrated to be responsive to APAP exposure.

## Discussion

Our microfluidic chip was designed to mimic the microstructure of the liver and enable more physiological culture conditions than other previous devices. Indeed, as well as achieving the 3D culture of hepatocytes, our architecture also allowed the cells to spatially organise into hepatocyte cords with in vivo like dimensions. Compared to previous studies describing the same microarchitecture, we were able to culture and analyse a larger number of hepatocyte cord-like structures on a single device^17–19^. For the first time, this architecture was then used to culture human HepaRG cells. We chose this hepatic cell line because they share a larger number of metabolic and functional similarities with human primary hepatocytes than the HepG2 and HuH7 cell lines, for instance. Compared to primary hepatocytes, HepaRG cell line offers an almost unlimited stock of hepatocytes of constant quality. Because these cells are widely used at two different differentiation stages^21–26^, our approach providing a single but versatile microfluidic tool for their culture, differentiation and maturation, is of crucial importance in applications targeting drug screening and toxicity. Rather than adjusting the biological and chemical conditions, additional and innovative physical modifications were made to render the device compatible with the seeding and culture of either proliferative or differentiated HepaRG cells. Thus, the geometry, resistivity and flow rate of our device were optimised by taking account of the migratory and adherence properties of these two cell types.

The ability to maintain cells within the chambers was cell-type dependent: distinct behavioural differences were evidenced in the case of the HepaRG cell line, depending on their level of differentiation. At an hepatoblast-like stage, these cells exhibited strong migratory and deformability capacities. The migratory features of hepatoblasts are indeed observed in vivo in the embryonic development of the liver^30^. But as this cell line originated from an hepatocarcinoma, they might also result from their cancerous origin. The capacity for deformation of cancerous cells is commonly characterized by their ability to pass through very thin microchannels^31^. In all cases, channels with a diameter smaller than 5 × 2 µm^2^ were shown to reduce their migration capacity^32^ and dimensions smaller than 10% of the cell nuclear cross section to eventually stop it^33^.These results guided our choice of slit dimensions to prevent cells from migrating through them. This phenomenon was not evidenced in the case of primary hepatocytes^17,18^. Indeed, the latter did not proliferate and because of their differing rigidity and size, behaved unlike the human cell lines in our chip. In the case of HepG2 cell culture in the same architecture with 5 × 2 µm^2^ slits, their migration out of the culture chamber could be evidenced but to a lesser extent as cells were not reported to proliferate inside the medium channel^19^. Although cells were better retained within the chambers using 2 × 2 µm^2^ slits, cytoplasmic extensions were still growing towards the medium channel. By using more complex slit networks, including a 3 × 2 µm^2^ central channel and 90° angles, we expected cells to be more prone to elongate into this wider central channel rather than going further up to the medium channel. Previous studies had indeed reported that between two microchannels, cancerous cells would rather enter in the wider one^34,35^. However, it appeared that the chemotactic recruitment of cells by the culture medium had a stronger impact on HepaRG cells than mechanical constraints. Unlike proliferative HepaRG cells, already differentiated HepaRG-hepatocytes were highly sensitive to the variations in pressure caused by manipulation of the chip. This was most likely due to their preferential adherence to each other rather than to the culture substrate of collagen-coated glass and PDMS. Indeed, the weaker adherence of HepaRG hepatocytes to the substrate compared to proliferative cells was evidenced in a different structure of microfluidic chip^29^. The addition of external capillary tubes with high hydraulic resistance offered an efficient alternative to desensitise the microfluidic device to pressure disturbances^36,37^. Finally, our system was suitable for the long-term culture of HepaRG cells loaded at both differentiation stages.

In our device, HepaRG cells could be maintained with a good viability for longer periods of time than other similar systems^14,17–19,29,38^. In order to assess the differentiation status of HepaRG cells in the device, we measured the albumin secreted in the medium channel during the culture, performed DCFA excretion test, and finally halted the experiment by fixing the cells and performing immunofluorescence labelling associated with confocal microscopy imaging. Thanks to the geometry of the device, and particularly the 40 µm height of the chambers, the formation of cord-like structures in 3D was demonstrated for the first time in this kind of chip reproducing liver microarchitecture. The confocal imaging of cells inside the chambers evidenced the aggregation of stacks of 3 to 4 cells in height and width. These observations were made at a greater degree of details than for most existing devices. In addition, these cord-like structures possessed functional MRP2 transporters on the apical side of polarized hepatocytes as evidenced by DCFA excretion. The multiplexing of chambers on a single chip provided a significant advantage as compared to the previously described one-chamber systems^17–19^ as the amount of the albumin produced by hepatocytes could be measured. As early as two days after the loading of HepaRG cells at either differentiation stages on chip, albumin secretion was detected, and then kept constant for up to 14 days. HepaRG cells loaded as hepatoblasts had spontaneously differentiated into hepatocytes, while already differentiated HepaRG hepatocytes had maintained their differentiation status. Moreover, we evidenced that differentiated cells loaded in our chip were sensitive to a low but chronic dose of APAP, with cell viability being reduced and the loss of intercellular tight-junctions.

The microfluidic system that we have developed and optimised is suitable for the long-term culture and differentiation of human hepatocytes, and well adapted to the different migratory and adhesion properties of these cells when seeded at their different stages of differentiation. In this device, hepatocytes take on a 3D organisation and can be cultured under flux for long periods of time. Using this microfluidic chip with hepatocytes from different sources could serve as a new platform for drug toxicity assays.

## Materials and methods

### Manufacture of the device

The device is made of PDMS using a conventional soft lithography technique^39^. The process is detailed in the Supplementary information and in the Supplementary Table S2.

### HepaRG™ cell culture

Cells were supplied by Biopredic International and cultured according to their instructions. They were used between passages 15 and 18. The cells were plated at a density of 2 × 10^4^ cells/cm^2^ and cultured with William’s E medium supplemented with L-glutamine (2 mM) and HepaRG^®^ Growth Medium Supplement with antibiotics (ADD710, Biopredic International, Saint-Grégoire, France). The medium was changed every two to three days. The cells were allowed to reach confluence before they started spontaneous differentiation. After 14 days of culture, the cells were ready to be detached and loaded onto the chip as “proliferative” or “hepatoblast-like” cells. To further differentiate cells towards hepatocytes on culture wells, the supplement was gradually changed to the HepaRG^®^ Differentiation Medium Supplement with antibiotics (ADD720) containing 1.7% DMSO over a two-to-three-day period.

### Selective detachment of hepatocytes derived from HepaRG cells

After culture for a total of 28 days, hepatocytes were selectively detached and loaded onto the chip as “differentiated” or “hepatocyte-like” cells. This previously described technique was intended to preferentially collect hepatocytes from the two cell populations with distinct morphologies that are present during a HepaRG cell culture^40^. Clusters of small refringent cells correspond to hepatocytes and large and spreading cells correspond to cells committed to a biliary fate. The technique consists in incubating them with trypsin–EDTA (0.05%) for 90 s at 37 °C, then gently flushing the plates with trypsin to selectively recover small clusters of hepatocytes.

### Loading and fluidisation protocol

First, the chips were sterilized with ethanol (70%) for 30 min, rinsed with PBS and then coated with collagen I from rat tail (50 µg/ml, C3867, Sigma-Aldrich, Saint-Louis, MI, USA) overnight at 4 °C. They were then filled with culture medium. To maintain differentiated HepaRG cells inside the chambers, as many fluid connections to the chip as possible were made before cell loading. All elements were autoclaved or sterilized with ethanol (70%). The main tubing used was made up of Teflon PFA (0.05 mm internal diameter (ID), 1/16″ outer diameter (OD)) and connected to the chip through wider tubes (silicone, 1 mm ID, 3 mm OD). A syringe filled with cell culture medium and an outlet tube to waste were both connected to one end of the medium channel. An external resistance was inserted between the syringe and the chip. This resistance was a capillary tube with a diameter of 25 or 50 μm and 10 or 20 cm long (tube references 62,510 or 65,020 respectively, from Cluzeau Info Labo, Sainte-Foy-La-Grande, France). 10 µl of a 1 × 10^7^ cells/ml solution were deposited into the cell channel outlets. To load the cells, suction was applied through the previously connected outlet tube through a negative pressure controller (MFCS™-EZ, Fluigent, Kremlin-Bicêtre, France; Supplementary Fig. S8) at − 25 mbar for the 25–5 µm device, − 35 mbar for the 25–2 µm device, and − 20 mbar for the 40–2 µm device. Chamber filling was monitored under a microscope. Once all the chambers had been loaded with cells, the suction was slowly turned off and the controller was disconnected from the chip. The ends of the cell channel were connected to waste. The chip and waste container were placed in the incubator and the syringe on the syringe pump (Low Pressure Syringe Pump neMESYS 290 N, CETONI, Korbußen, Germany). A flow rate of 375 nl/min was applied after 4 h of static conditions. The medium in the syringe was changed every 3 to 4 days.

### Cell viability assay

Two drops of the NucBlue™ Live ReadyProbe™ reagent (R37605, Thermofisher Scientific, Waltham, MA, USA) were diluted into 1 ml of cell culture medium without red phenol and injected inside the chip for 2 h at 375 nl/min. Then the chip was infused with a solution of fluorescein diacetate (10 µg/ml) and propidium iodide (5 µg/ml) for 1 h and finally washed with William’s E medium for 30 min. As positive control and to set up the microscope parameters, the chip was first treated with ethanol (70%) and washed with William’s E for 30 min each. Images were recorded with an EVOS™ FL Auto Imaging System M7000 (Thermofisher Scientific). Cell viability was calculated as the ratio of propidium iodide negative cells divided by the total number of nuclei inside the chambers.

### Bile canaliculi functionality

5(6)-carboxy-2’,7’-dichlorofluorescein (DCFA) (5 µM; AB145439, Abcam, Cambridge, UK) in William’s E medium without red phenol was injected inside the chip. Stacks of images were recorded with an EVOS™ FL Auto Imaging System and deconvolved as detailed in the Supplementary information.

### Cell staining and observation

All dilutions were performed in phosphate buffered saline (PBS) 1X. The lengths of all steps are detailed in Supplementary Table S3. After the culture period, the cells were fixed on the chip with PFA (4%). They were then permeabilised using a solution containing bovine serum albumin (BSA, 1% w/v), EDTA (5.37 × 10^−1^µmol/L), and triton-X-100 (0.1% v/v). They were blocked with BSA (3% w/v) and incubated overnight at 4 °C with primary antibodies diluted in BSA (1% w/v) (Supplementary Table S4). The cells were then washed with a solution of tween 20 (0.1% v/v), and incubated at room temperature for 2 h with secondary antibodies diluted in BSA (1% w/v) (Supplementary Table S4). They were washed again with the tween solution and finally with milliQ water. Fluorescent images were recorded using either an EVOS™ FL Auto Imaging System or a Leica SP8 confocal microscope and analysed using Fiji software^41,42^, as detailed in the Supplementary information.

### Human albumin secretion quantification

To measure the quantity of albumin produced on chip, the medium going out of the device by the medium channel was collected every 4 h at different days of culture and stored at − 80 °C. An enzyme-linked immunosorbent assay for the quantification of human albumin was used according to the manufacturer’s instructions (E80-129, Bethyl laboratories, Montgomery, TX, USA).

### Toxicity assay

Chips were loaded with differentiated HepaRG cells and cultured for 14 days in differentiation medium under flux. They were then exposed for 6 days to a solution of 2 mM APAP in the same medium that was previously used. The APAP was diluted from a perfusable solution at 10 mg/ml used in the clinic (CIP 34,009 576 910 73, Macopharma, Mouvaux, France). Syringes were changed every 3 to 4 days. Cell morphology inside the chambers were assessed by phase contrast imaging at day 0, 1, and 6 of treatment. At day 6, cell viability was assessed with NucBlue™ reagent and propidium iodide. The viability calculation process is detailed in the Supplementary information. The chips were then fixed and stained for ZO-1 and MDR3 markers, and imaged with an EVOS™ FL Auto Imaging System. Images were deconvolved as detailed in the Supplementary information. As 2D control, well plates containing 14-day differentiated cells were cultured for another period of 14 days (for a total of 28 days in differentiation medium). They were exposed to 0, 1, 2, 5, 10, 20, and 40 mM APAP for 24 h or 2 mM APAP for 6 h. Cell viability was assessed with NucBlue™ reagent and propidium iodide (15 min incubation at 37 °C), as detailed in the Supplementary information. The 6 h treated wells were fixed and stained for ZO-1 marker.

### Statistical analysis

All variables were expressed as means ± standard deviations. The comparison of cell viability on chip after 6 days of APAP treatment or no treatment was performed by a Student’s t test. A p value of less than 0.05 was regarded as statistically significant.

### Simulations

A 3D COMSOL Multiphysics^®^ model (COMSOL AB, Stockholm, Sweden) was used to estimate the distributions of velocity and shear stress within the device. Twenty chambers in series and containing no cells were modelled. We chose a creeping flow rate for water at 37 °C coupled with the transport of dilute species for oxygen. A flow rate was imposed at one inlet of the medium channel. Pressure was set at 0 Pa for the three other outlets. The oxygen consumption rate was set at q_O2_ = 5 × 10^–17^ mol/s/cell and Michaelis–Menten kinetics were used to describe the reactions within the chambers^11,43–47^. To simulate tissue-loaded chambers, different values of this gas diffusion coefficient were investigated. These simulations are detailed in Supplementary information.

## Supplementary Information

Supplementary Information.

## Data Availability

The datasets generated during and/or analysed during the current study are available from the corresponding author on reasonable request.
