# Supplementary material for: A versatile microfluidic tool for the 3D culture of HepaRG cells seeded at various stages of differentiation
Source: Sci Rep. 2021 Jul 7;11:14075. doi: 10.1038/s41598-021-92011-7 (PMC8263583; doi:10.1038/s41598-021-92011-7)
Supplement: Supplementary file 1 — Supplementary Information. [file 41598_2021_92011_MOESM1_ESM.pdf]

Supplementary information

A versatile microfluidic tool for the 3D culture of HepaRG cells seeded at various stages of differentiation

Manon Boul[1,2,3,4,5], Nassima Benzoubir[2,4], Antonietta Messina[2,4], Rasta Ghasemi[5], Ismail Ben Mosbah[6], Jean-Charles Duclos-Vallée[2,4,7], Anne Dubart-Kupperschmitt[2,4], and Bruno Le Pioufle[*3,4,5]

[1]Université Paris Saclay, ENS Paris Saclay, CNRS SATIE, 4 avenue des Sciences, F91190 Gif-sur-Yvette, France
[2]UMR_S 1193 INSERM/Université Paris-Saclay, F94800 Villejuif, France
[3]Université Paris Saclay, ENS Paris Saclay, CNRS LUMIN, F91190 Gif-sur-Yvette, France
[4]FHU Hépatinov, Centre Hépato-Biliaire, Hôpital Paul Brousse, F94800 Villejuif, France
[5]Université Paris Saclay, Institut d'Alembert, ENS Paris Saclay, CNRS, F91190 Gif-sur-Yvette, France
[6]Biopredic International, Parc d'Affaires La Bretêche, 35760, Saint-Grégoire
[7]APHP, Centre Hépato-Biliaire, Hôpital Paul Brousse, F94800 Villejuif, France
*Correspondence and requests for materials should be addressed to B.L-P. (email: bruno.le-pioufle@ens-paris-saclay.fr)

Efficiency of the cell loading procedure on chip

A negative pressure controller was used to load cells as it provides a smooth way to move them into the chambers and has a faster response time than a syringe pump. In addition, the suction decreases as cells fill the chambers and block slits because the chamber resistivity increases.

During the loading procedure of cells on the device (depicted in the **Supplementary Fig. S4,** with the negative pressure controller set to -20 mbar and connected to the 40-2 µm device), analytical calculations evidenced that the suction experienced by cells differed by 30% between empty chambers: the pressure difference at the extremities of the chambers decreased from -17 mbar in the chambers that are closer to the suction to -13 mbar for the ones near the syringe connection. Experimentally, cells filled the chambers homogeneously throughout the chip. The number of cells injected into the chip and required for proper aggregation was standardized at 10^5 cells (10 µl of a 1.0×10^7 cells/ml solution deposited at the cell channel outlet). Cell nuclei were counted on two chips of the 40-2 µm device after 2 days in static conditions. The average number of cells inside one chamber was 210 ± 39, leading to a 19 % inhomogeneity between chambers. By dividing the chambers into 4 groups (from the 1st to the 5th chamber, the 6th to the 10th, the 11th to the 15th and the 16th to the 20th), no significant difference in the average of nuclei count was found (**Supplementary Table S1**). The filling of the chips was thus controlled and homogeneous.

Chamber n°	Mean nuclei number inside one chamber	Standard deviation
1 to 5	223	42
6 to 10	207	33
11 to 15	210	45
16 to 20	201	35

Supplementary Table S1. Mean number of cell nuclei inside the chambers of a 40-2 µm device, after two days in static conditions, which were divided into 4 groups (n = 2 chips).

Cytoplasmic extensions in the 25-2 µm device

Supplementary Figure S1. Evidence for the cytoplasmic extensions of HepaRG cells loaded as proliferative, after two days under static conditions, evidenced by fluorescent staining (blue: DAPI, green: phalloidin). Images were obtained with a confocal microscope. Scale bars = 40 µm.

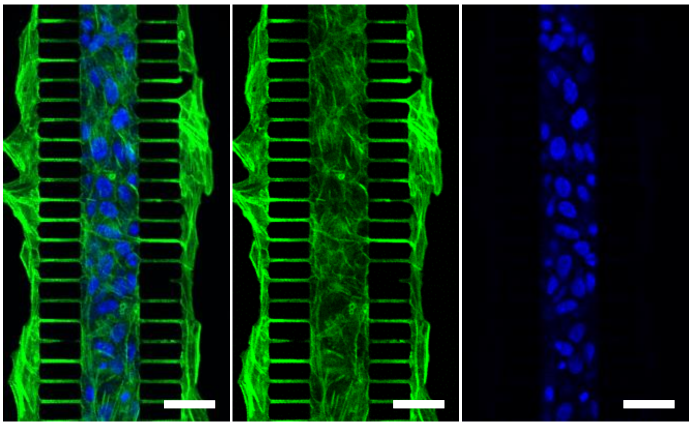

Discontinuous slit design

Supplementary Figure S2. Design of the slits presenting 90° angles. Two arrays of 2x2x17 µm³ slits are disposed alternatively on both sides of a central 2x3 µm² channel. **a**) Zoom on one chamber. **b**) Zoom on the dead-end portion of a chamber. Scale bars = 37 µm.

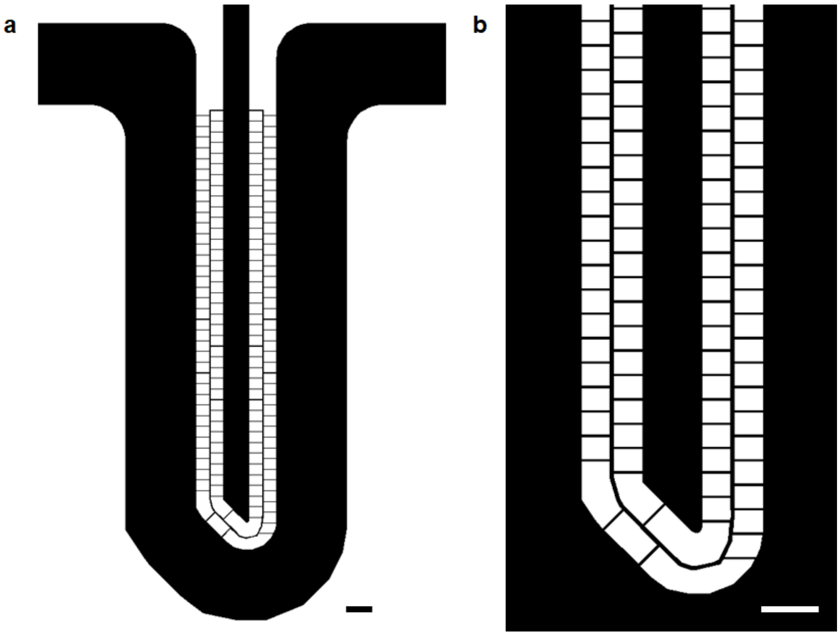

Detailed simulation method and additional results

Using COMSOL® software, we produced a 3D model of an array of 20 chambers in series with straight slits from the 40-2 µm device. The physical presence of cells was not taken into account. Water flowed within this structure at a temperature of 37°C and a creeping flow mode was chosen. A half flow rate of 400 nl/min was imposed at one inlet to the medium channel. The pressure was set at 0 Pa for the three outlets. Due to the computational time necessary for this 3D model to converge, the transport of oxygen could not be calculated under this simulation. A simpler model of one 3D chamber was then used. The values for fluid velocity and pressures at the input and outputs of the first, tenth, and twentieth chambers in the full simulation were taken and used in this new model. The transport of dilute oxygen species was coupled to the creeping flow mode. A block of material assumed to be PDMS surrounded the chamber and rose above it by 4 mm. The initial oxygen concentration was set at 0.2 mol/m^3 in both the fluid and PDMS and the diffusion coefficient was set at 1.8x10^{-9} m^2/s in water[1–4] and 3.25x10^{-9} m^2/s in PDMS[5,6]. The concentrations of oxygen at fluid input and at the surface of the PDMS the most distant from the circuit were kept at a constant value of 0.2 mol/m^3. Oxygen was consumed at a rate of q_{O2} = 5x10^{-17} mol/s/cell through a Michaelis-Menten equation[1,2,7] defined in the volume of the chamber, from its dead-end portion at -50 µm to its exit at 950 µm along its y axis (**Supplementary Fig. S4**): volumetric oxygen consumption rate in a chamber $OCR = \frac{[O_2] \times Nc \times q_{o2}}{Vch\ (Km + [O_2])}$, with [$O_2$], the oxygen concentration at a given point of the mesh[5,8–12]. The Michaelis constant Km value was set at 0.005 mol/m^3. The number of cells (Nc) within a chamber was set at 224 cells and the volume of the chamber (Vch) was 1.41x10^{-12} m^3.

A flow rate range suitable for the culture of hepatocytes was determined by simulations. Analytical calculations showed that chambers with straight or discontinuous slits would have equivalent hydraulic resistivity (absolute difference of 2%), so simulations were performed on arrays of straight slits. During numerical simulations of the device, the presence of cells inside the chambers was not taken into account despite the fact that this might reduce fluid velocity. Our calculations therefore corresponded to the worst conditions considering the shear stress applied to cells. To determine the maximal flow rate value, the mapping of fluid velocity and shear stress inside the chambers was analysed. For an inflow of 400 nl/min, the mean fluid velocity inside the chambers was 5.53x10^{-5} m/s, one order of magnitude lower than in the medium channel (3.57x10^{-4} m/s). A fluid velocity gradient was evidenced within the chambers, while variations in velocity were also observed between successive chambers (**Supplementary Fig. S3**). The flow velocity gradient inside the chambers was quantified along the y axis from their dead-end portion - connected to the medium channel through the slits – towards the outlet from the chamber - connected to the cell channel. Velocity was found to increase linearly with y in the dead-end portion of the chamber and reach a plateau at its outlet (**Supplementary Fig. S4**). Average fluid velocity also varied between the chambers, different from the first to the last reached by the medium (chambers 1 and 20, respectively), falling by around one order of magnitude. The same trends and differences were also evidenced with respect to the shear stress values calculated on the surfaces of the chambers (**Supplementary Fig. S4**). These values were locally higher at slit/chamber interfaces but remained lower than 0.1 Pa on average, in the knowledge that 0.5 Pa is the maximum acceptable shear stress for hepatocytes[13]. Thus, on the device, the higher shear stress value that needed to remain below 0.5 Pa was that found at the exit from the first chamber. In

addition, the mean shear stress inside a chamber increased linearly with the flow rate. As a result of this, the maximum flow that could be imposed was 9.5 µl/min.

To determine the minimal flow that would keep the cell alive, we estimated the level of their oxygen supply, which was found to arise from its diffusion through PDMS: independently of the flow rate applied, the concentration of the oxygen supplied to cells was above the hypoxic limit, considered to be 0.04 mol/m^3 (equivalent to 32 mmHg)[7,14,15] in all chambers (**Supplementary Fig. S5**). Indeed, The diffusion coefficient of oxygen is at least 1.6 times higher through PDMS (3.25x10^{-9} m^2/s)[5,6] than through the slits and tissue (evaluated from 3.4x10^{-10} to 2.0x10^{-9} m^2/s in the literature[1,2], as compared to 2.69x10^{-9} m^2/s in the culture medium[3,4]).

In the case of tissue-loaded chambers, the flow behaviour would be affected and we can reasonably assume it would lower it. Cells would therefore experience a lower shear stress, which is beneficial to tissue forming. In an extreme situation, the oxygen supplied to cells would be limited to its diffusion through the slits, chambers and PDMS (no convective flow). The compaction of cell aggregates in the chambers would lower the diffusion coefficient of oxygen. The oxygen concentration in chambers containing cells was thus simulated using a low values of diffusion coefficient taken in the range 3.4x10^{-10} - 2.0x10^{-9} m^2/s[1,2], in static conditions (**Supplementary Fig. S6**). Oxygen concentration was measured in the middle of the chamber (red line in **Supplementary Fig. S4**) at the glass surface (which is the surface the further away from the PDMS boundary). The diffusion of oxygen from the medium and through the slits and chamber only resulted in hypoxic conditions for cells. When considering the contribution of PDMS, the oxygen concentration was far above the hypoxic limit even for the smallest value of diffusion coefficient. Thus, the worst conditions for cells are (i) for the shear stress, the one described with empty chambers in the **Supplementary Fig. S3**, (ii) for the oxygen supply, the **Supplementary Fig. S6** with the lowest value of its diffusion coefficient.

Supplementary Figure S3. Numerical modelling of the culture conditions for cells on the 40-2 µm chip. 3D COMSOL® simulation of 20 chambers in a series with straight slits, without any cells, at 37°C. The flow rate at the inlet (symbolized by black arrows) is 400 nl/min. At the three outlets the pressure is set at 0 Pa. **a**) Fluid velocity on the xy plan (z = 20 µm) for chambers 1, 10, and 20. Scale bar = 100 µm. **b**) Shear stress on the xy plan (z = 0 µm). Left-hand side: general view of the first chamber, scale bar = 100 µm. Right-hand side: zoom on the dead-end portion of chambers 1, 10, and 20. Scale bar = 40 µm. **c**) Table of the mean values for velocity and shear stress inside chambers 1, 10, and 20.

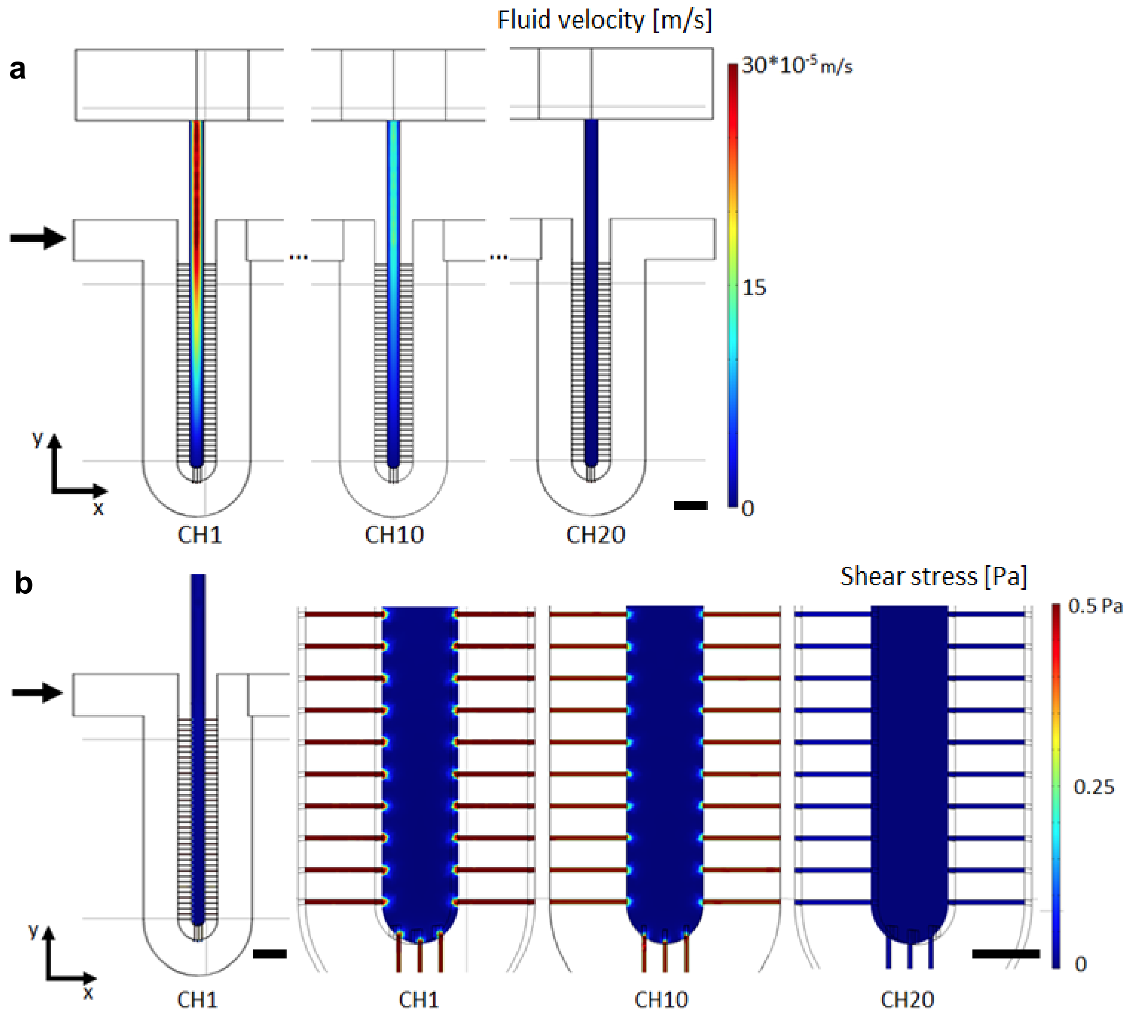

c

400 nl/min	Mean velocity [m/s]	Mean shear stress [Pa]
**40-2 device**		
Chamber n°1	9.08e-5	2.1e-2
Chamber n°10	3.98e-5	9.0e-3
Chamber n°20	1.97e-5	4.0e-4

Supplementary Figure S4. Fluid velocity and shear stress inside chambers of the 40-2 µm device for a flow rate of 400 nl/min. **a**) Diagram of one chamber. Velocity and shear stress values were measured in the middle of the chamber, along its y axis, from -50 µm to 950 µm (red line). **b**) Evolution of fluid velocity values in the middle of the chamber along its y axis (z = 20 µm) for chambers 1, 10, and 20. **c**) Evolution of shear stress values in the middle of the chamber along its y axis (z = 0 µm) for chambers 1, 10, and 20. **d**) Zoom on the evolution of the shear stress values shown in c) when the maximum shear stress was set at 0.1 Pa.

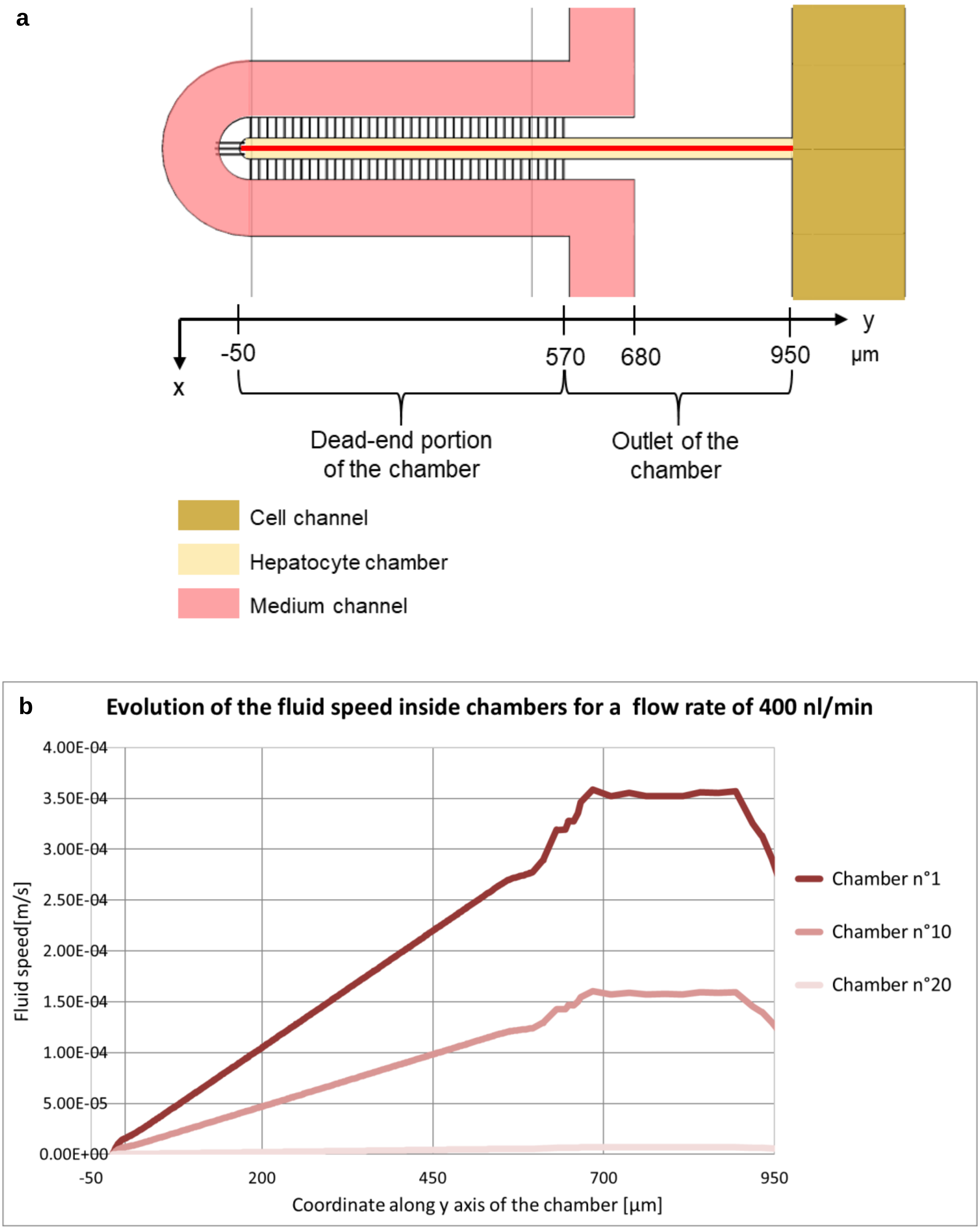

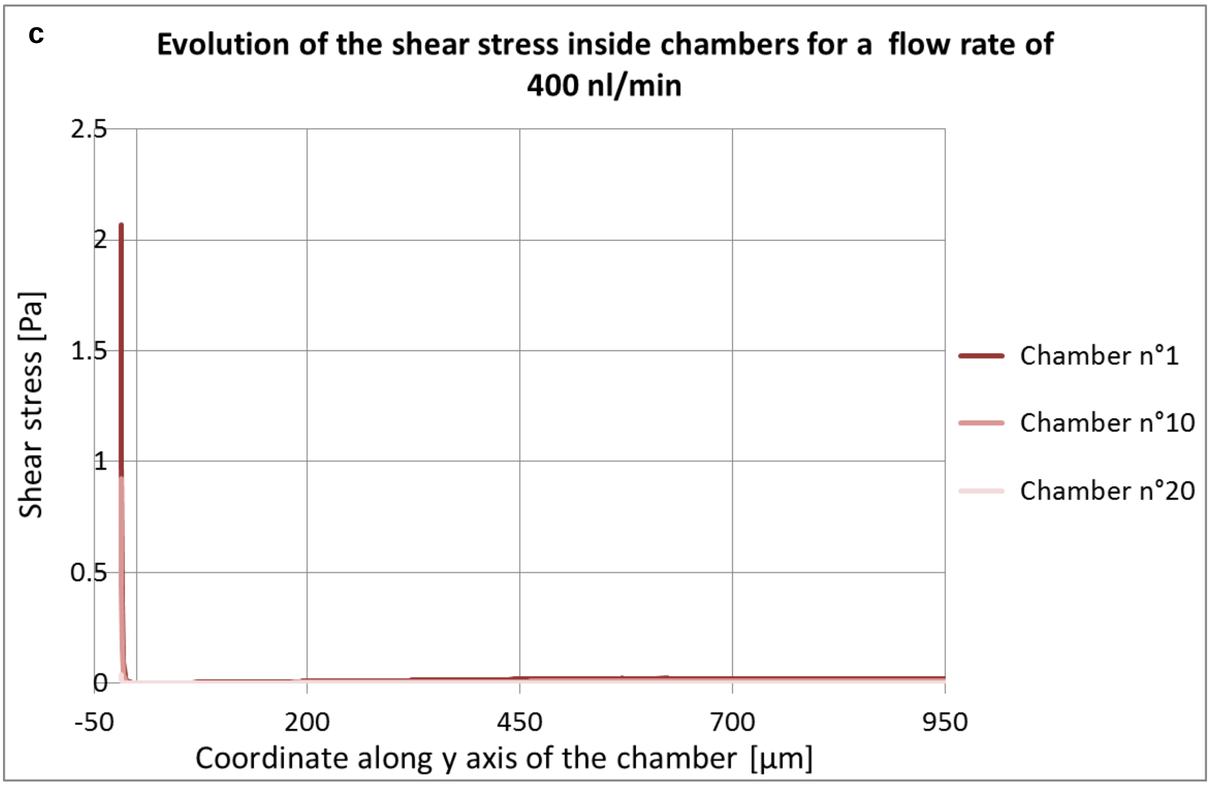
c
Evolution of the shear stress inside chambers for a flow rate of 400 nl/min
Shear stress [Pa]
2.5
2
1.5
1
0.5
0
-50
200
450
700
950
Coordinate along y axis of the chamber [µm]
Chamber n°1
Chamber n°10
Chamber n°20

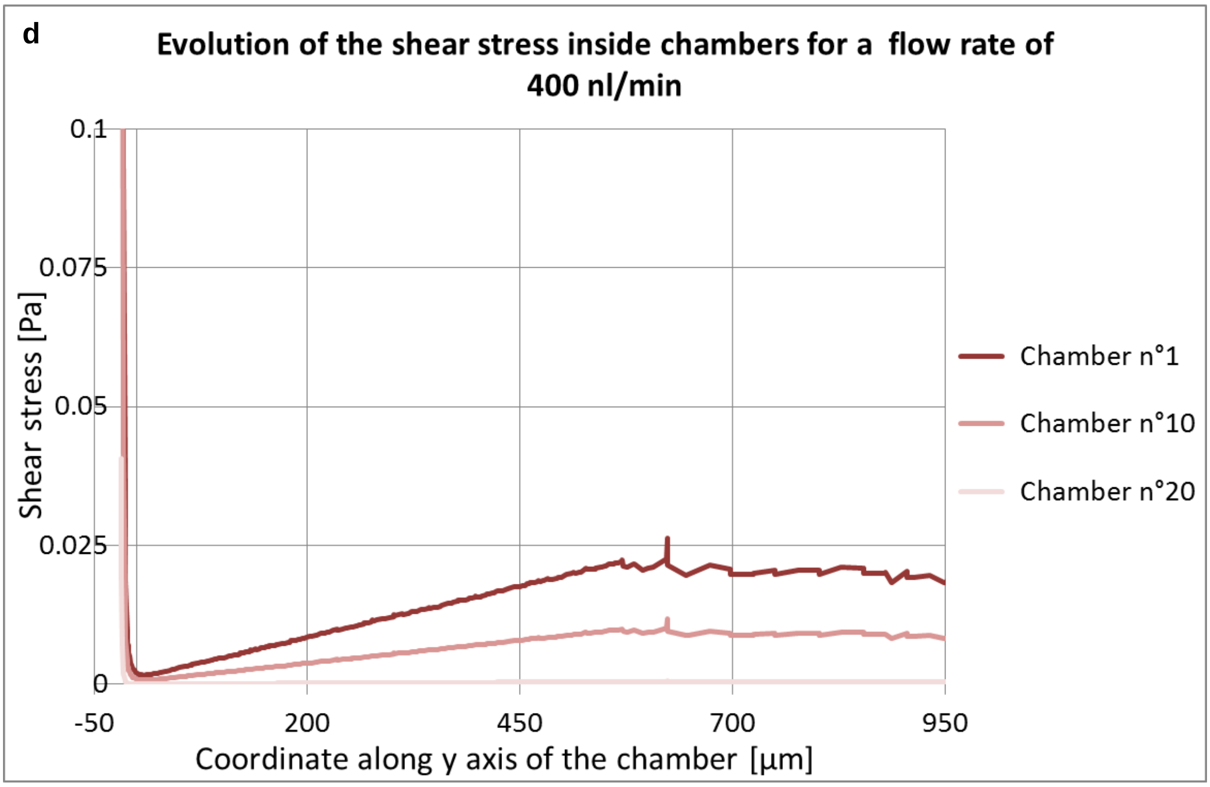
d
Evolution of the shear stress inside chambers for a flow rate of 400 nl/min
Shear stress [Pa]
0.1
0.075
0.05
0.025
0
-50
200
450
700
950
Coordinate along y axis of the chamber [µm]
Chamber n°1
Chamber n°10
Chamber n°20

Supplementary Figure S5. Evolution of oxygen concentrations in the middle of the chamber with the 40-2 µm design. The diffusion coefficient of oxygen was set to $1.8x10^{-9}$ m^2/s in the slits and chamber. The following cases are represented: first, oxygen is only diffusing from the medium channel input through the liquid (*No fluid convection, without diffusion through PDMS*). Then, a flow rate of 400 nl/min is set and oxygen is assessed in the different chambers (*Chambers 1, 10, and 20 without diffusion through PDMS*). Finally, the contribution of oxygen diffused through PDMS is added with either no flow or a 400 nl/min input flow (*No fluid convection + diffusion through PDMS* or *Chamber n°1 + diffusion through PDMS*, respectively).

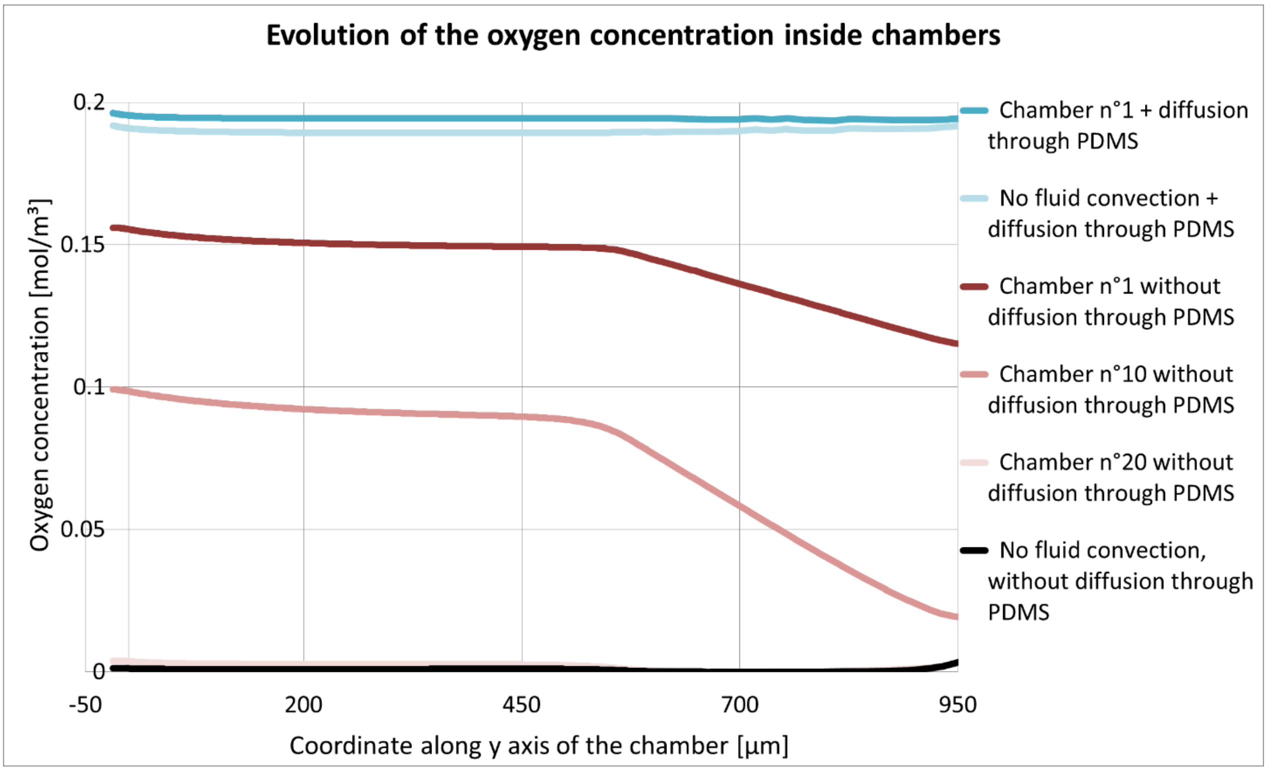

Supplementary Figure S6. Oxygen concentration in static conditions in the middle of the chambers (z = 0 µm) along the y axis of the chamber. The gas is diffusing through the slits and chambers for different values of its diffusion coefficient (D = 3.4×10^{-10} or 2×10^{-9} m^2/s), and from the PDMS (D = 3.25×10^{-9} m^2/s).

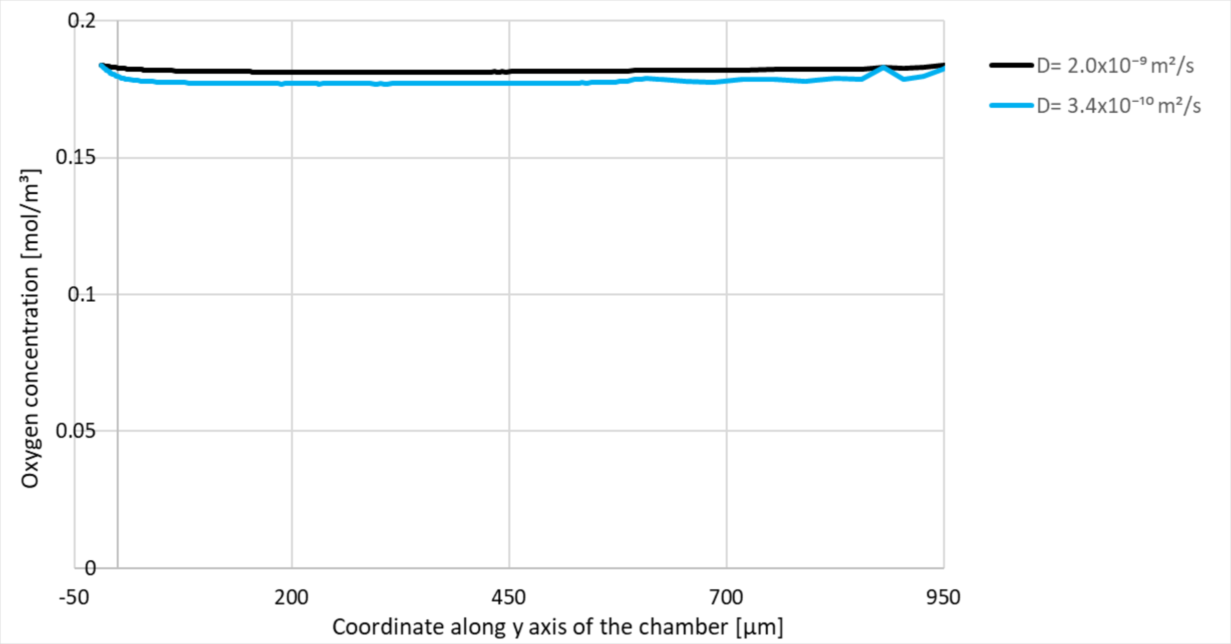

Desensitising the device to pressure disturbances

When culturing differentiated HepaRG cells inside the 40-2 µm device, they were expelled from the chambers by pressure disturbances (**Supplementary Fig. S7**). We calculated that a height difference of 20 cm between the syringe and the chip induced a flow of 4.6 µl/min within the medium channel. This flow was acceptable in terms of the shear stress induced when compared to the values obtained by the simulations but remained sufficient to expel poorly adherent cells from the chambers. To reduce unwanted flux, a capillary was inserted between the syringe and the chip (**Supplementary Fig. S8**). We tested two capillary tubes: the first with an internal diameter of 50 µm and a length of 20 cm, and the other with an internal diameter of 25 µm and a length of 10 cm. They were respectively 36 and 283 times more resistive than our entire circuit. The unwanted flux generated by pressure disturbances was thus reduced by the same factor. Because our devices were perfused with a syringe pump, these capillary tubes did not affect the repartition of fluid velocity within the chip for a given inflow rate. In addition, as many fluid connections to the chip as possible were made before cell loading.

To estimate the circuit resistivity, we used the electric–hydraulic analogy ΔP = R*Q, where ΔP is the pressure difference between the inlet and outlet, Q the flow rate and R the hydraulic resistance of the circuit. We divided the structure into a set of resistances and calculated their resistivities using the following approximation[16] : $R = \frac{12\,\mu\,L\,\alpha}{h\,(1-0.63\alpha)}$, α = h/w ≤ 1, with µ the dynamic viscosity of the fluid, L the length of the channel, w its width and h its height. The resistance network was modelled using TINA software (Texas Instrument, Dallas, TX, USA), with voltage sources at all outlets. By knowing the difference in voltage imposed and

evaluating the current generated, the resistance of the circuit was found to be 2.56×10^4 $Pa.s.mm^{-3}$ at 37°C. The resistivity of the capillaries used as external resistances was calculated as: $R = \frac{8\,\mu\,L}{\pi\,r^4}$, with μ the dynamic viscosity of the fluid, L the length of the capillary and r its radius.

Supplementary Figure S7. Strategy to adapt cell culture on chip to less adherent HepaRG hepatocytes in the 40-2 µm device. **a**) **i**) After loading onto the device, **ii**) the cells were expelled from the chambers when fluidisation tubes were connected to the chip or when the heights of the syringe and device were modified. **b**) Bright field images **i**) at day 0 and **ii**) at day 20 of cells being loaded and then cultured at 300 nl/min in a device that included external resistances. Scale bars = 100 µm.

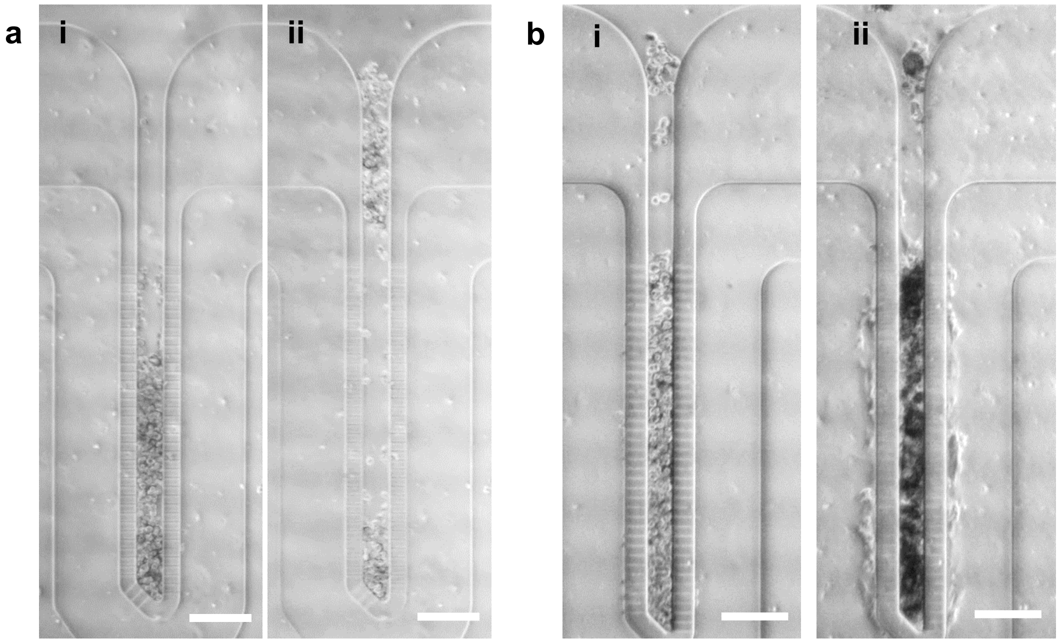

Supplementary Figure S8. Loading protocols. **a**) To maintain hepatocytes derived from HepaRG cells in the chambers, a medium-filled syringe connected to a hydraulic resistance (orange box) was connected to the medium channel before suction was started. At the other extremity of this channel, an outlet tube (red line) was also connected and suction was then started by connecting it to the pressure controller (blue arrow). Cells were deposited as previously described (green circles). **b**) View of the system adapted for HepaRG hepatocytes. The syringe containing the cell culture medium is connected to the chip via tubes. The medium leaving the chip is collected to waste. To reduce the sensitivity of the device to pressure variations and be able to maintain the cells within the chambers, external high hydraulic resistances (red arrows) were added between the syringe and chip.

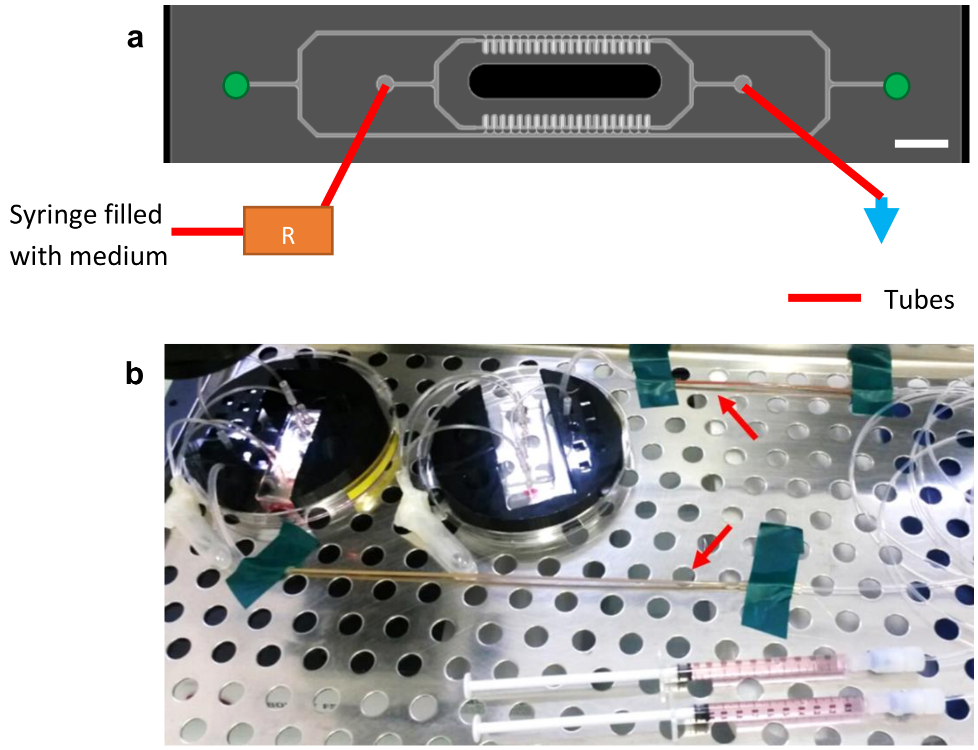

Treatment of fluorescent images

Fiji software[17] was used to adjust the brightness and contrast of each fluorescence channel of the images acquired. The different optimised images were then merged. Within the image stacks in the chambers, fluorophore intensities decreased when moving from the glass slide to the upper PDMS surface. To obtain more uniform sectional images along the length of the chambers, the Stack Contrast Adjustment plugin[18] was used.

To determine the number of nuclei in the chambers, the DAPI maximum intensities of stacks were projected on the same plan and the nuclei counted manually. The portion of the chamber in which the cells were counted was between -50 to 680 µm along the y axis (**Supplementary Fig. S4**).

To deconvolve stacks of images taken with an epifluorescent microscope, a theoretical point-spread function image was generated using the Diffraction PSF 3D plugin. Then, the DeconvolutionLab2 plugin[19] with 40 iterations of the Richardson-Lucy algorithm was used. The

image background was substracted with Fiji “Substract Background” tool (50 pixels) and the stack maximum or median fluorescent intensities were projected onto a single plan.

Evaluation of cell viability after their culture period on the chip

Supplementary Figure S9. Live and dead assay on chips loaded with **a**) proliferative or **b**) differentiated HepaRG, after 15 days of culture under a flux of 375 nl/min. Three chambers were imaged with epifluorescent or bright field microscopy. All nuclei were stained with the NucBlue^{TM} reagent (blue), live cells with fluorescein diacetate (green), and dead nuclei with propidium iodide (red). Scale bars = 100 µm.

a

b

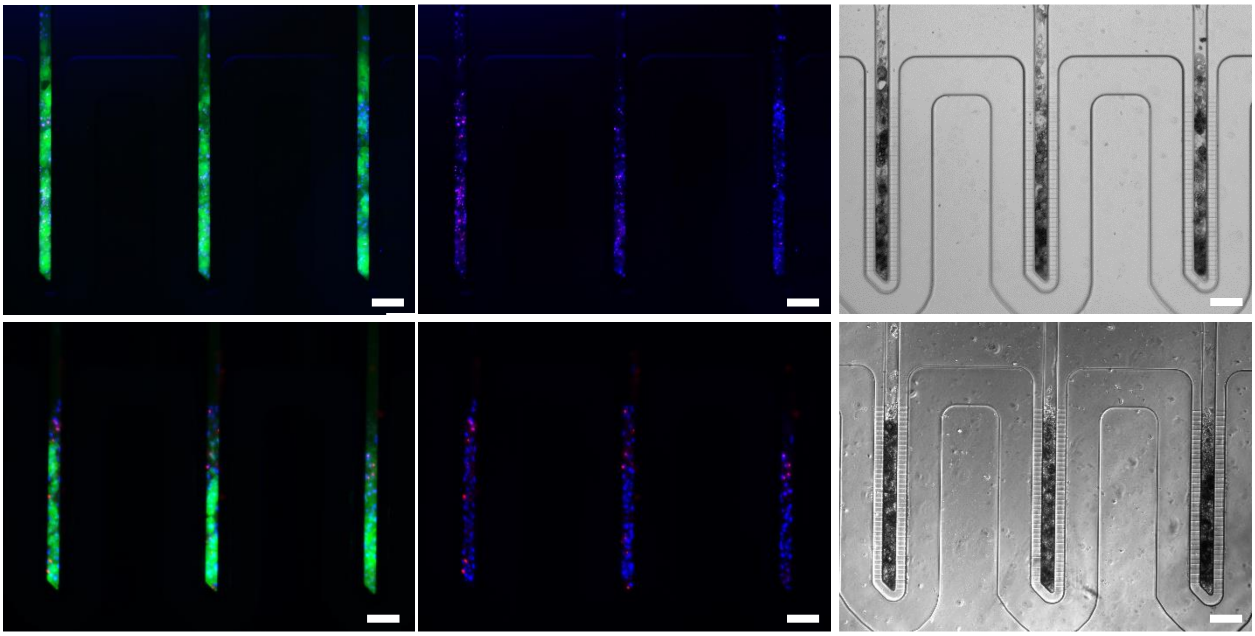

Acetaminophen treatment on chip and in 2D cultures

During the acetaminophen (APAP) treatment of chips, cells were evidenced to detach from their support and to be expelled from the chambers. In addition, even in chambers in which some aggregates could still be seen, the Live and Dead assay revealed that the majority of them were necrotic, with only a few nuclei that could be seen (N_{ch}). We therefore adapted our way to calculate cell viability. The filling of chamber surfaces with cells were compared at day 0 and 6 from phase contrast imaging (S_{d0} and S_{d6}, respectively). From the control chips, an average number of cells per chamber surface unit was calculated ($N_{ref/S}$). The number of dead cells was calculated as the sum of the propidium iodide positive cells, an estimation of the cells that detached between day 0 and 6 ((S_{d0} – S_{d6}) x $N_{ref/S}$), and an estimation of the necrotic cells at day 6 ($N_{ref/S}$ x S_{d6} - N_{ch}). It was divided by the total number of cells in the chamber which was the sum of the nuclei stained with NucBlue^{TM} reagent, the estimated number of detached cells, and the estimated number of necrotic cells. For 2D cultures, the number of dead cells was calculated as the sum of the propidium iodide positive cells and the number of detached cells. The latter was estimated as the difference between the average number of nuclei in control conditions and the number of nuclei in treated samples.

Supplementary Figure S10. Cytotoxic effect of acetaminophen after 24h on HepaRG cells cultured in well plates for 28 days in differentiation medium. **a**) Cell viability depending on the concentration of APAP to which cells were exposed during 24h (n = 2). **b**) Phase contrast and fluorescent imaging of cells exposed to 20 mM APAP for 24h and stained for dead cells with propidium iodide (red). Scale bars = 200 µm.

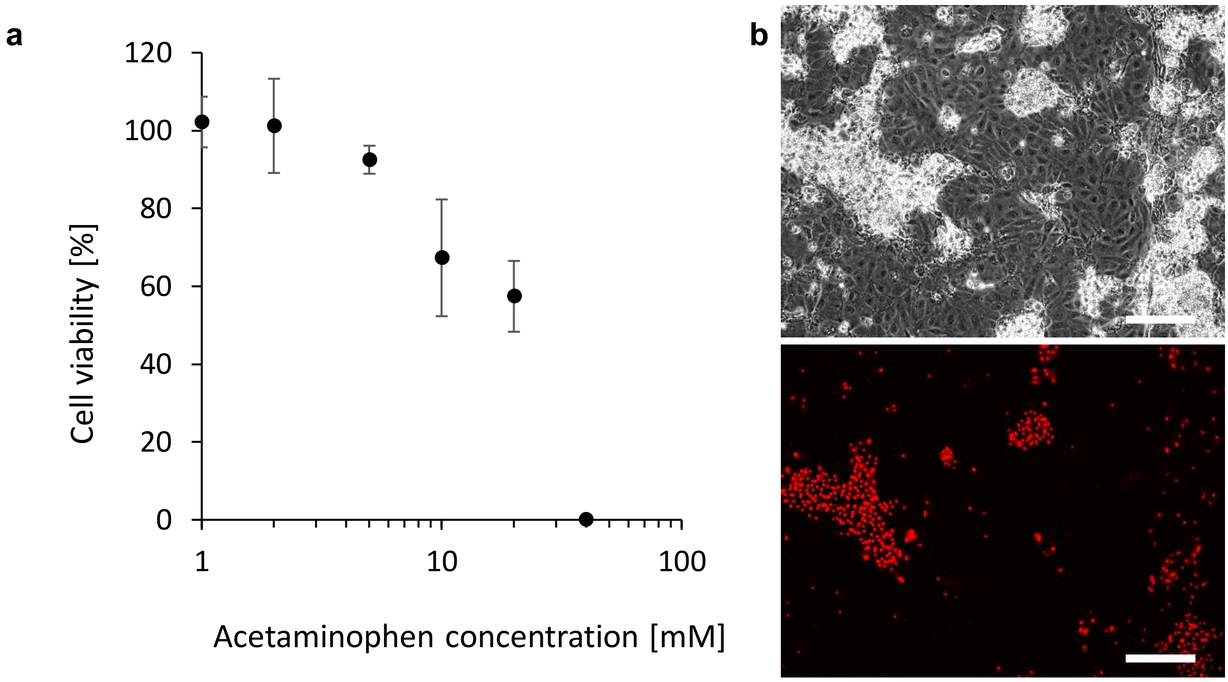

Supplementary Figure S11. Phase contrast imaging of chips loaded with differentiated HepaRG, cultured for 14 days and treated or not with 2 mM APAP for 6 days. **a**) Control chip at day i) 0, ii) 1, iii) 6. **b**) and **c**) Two different chambers from a chip treated with 2 mM APAP at day i) 0, ii) 1, iii) 6. Cell aggregates are dissociating (white arrow). Scale bars = 40 µm.

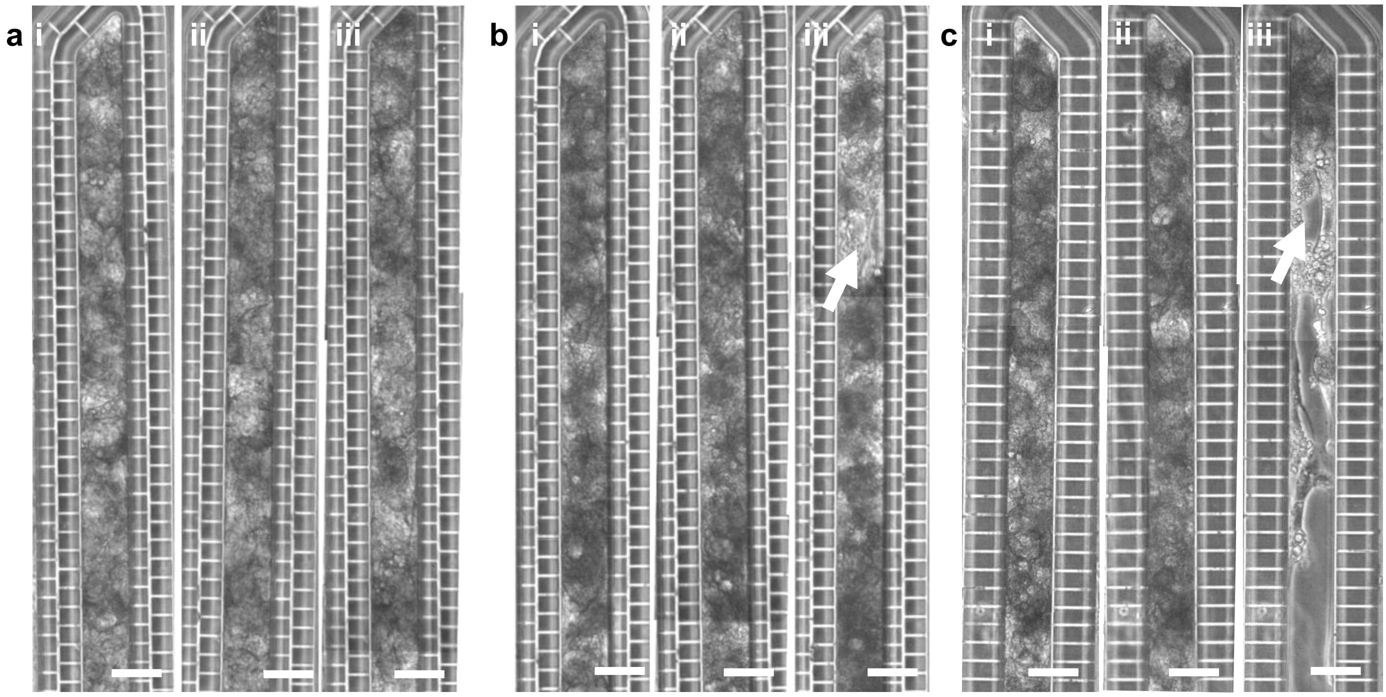

Supplementary Figure S12. Phase contrast and immunofluorescent imaging of HepaRG cells in well plates. They were cultured for 28 days in the differentiation medium, fixed and stained for nuclei (blue) and ZO-1 (red). **a**) Control cells. **b**) Cells treated with 2mM APAP for 6 hours. Scale bars = 75 µm.

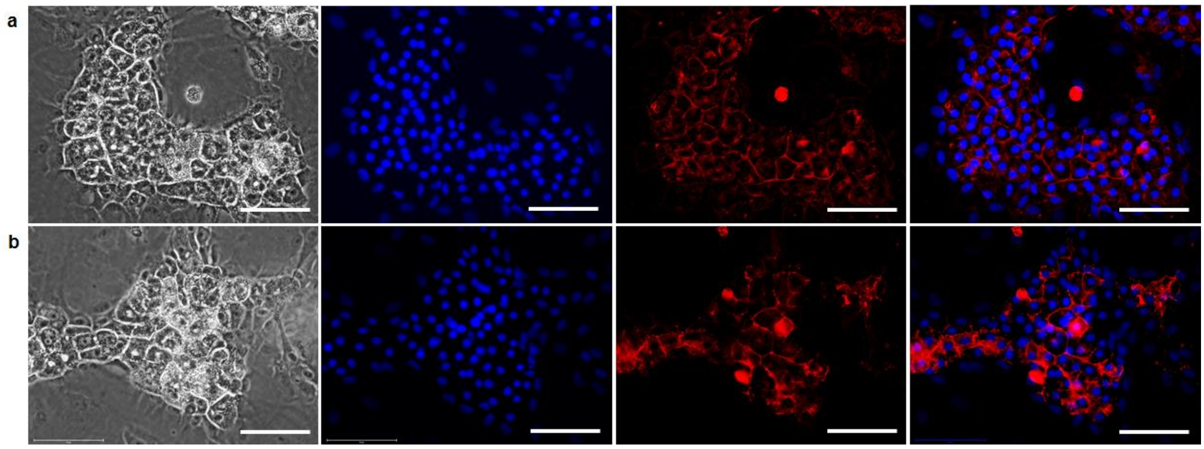

Supplementary Figure S13. Immunofluorescent analysis of differentiated HepaRG cells loaded, cultured on chip and exposed to APAP. Cells were fixed and stained for nuclei (blue), ZO-1 (green), and MDR3 (red). Image of 3 chambers from: **a**) a control chip, **b**) a chip exposed continuously to 2 mM APAP for 6 days. Scale bars = 100 µm.

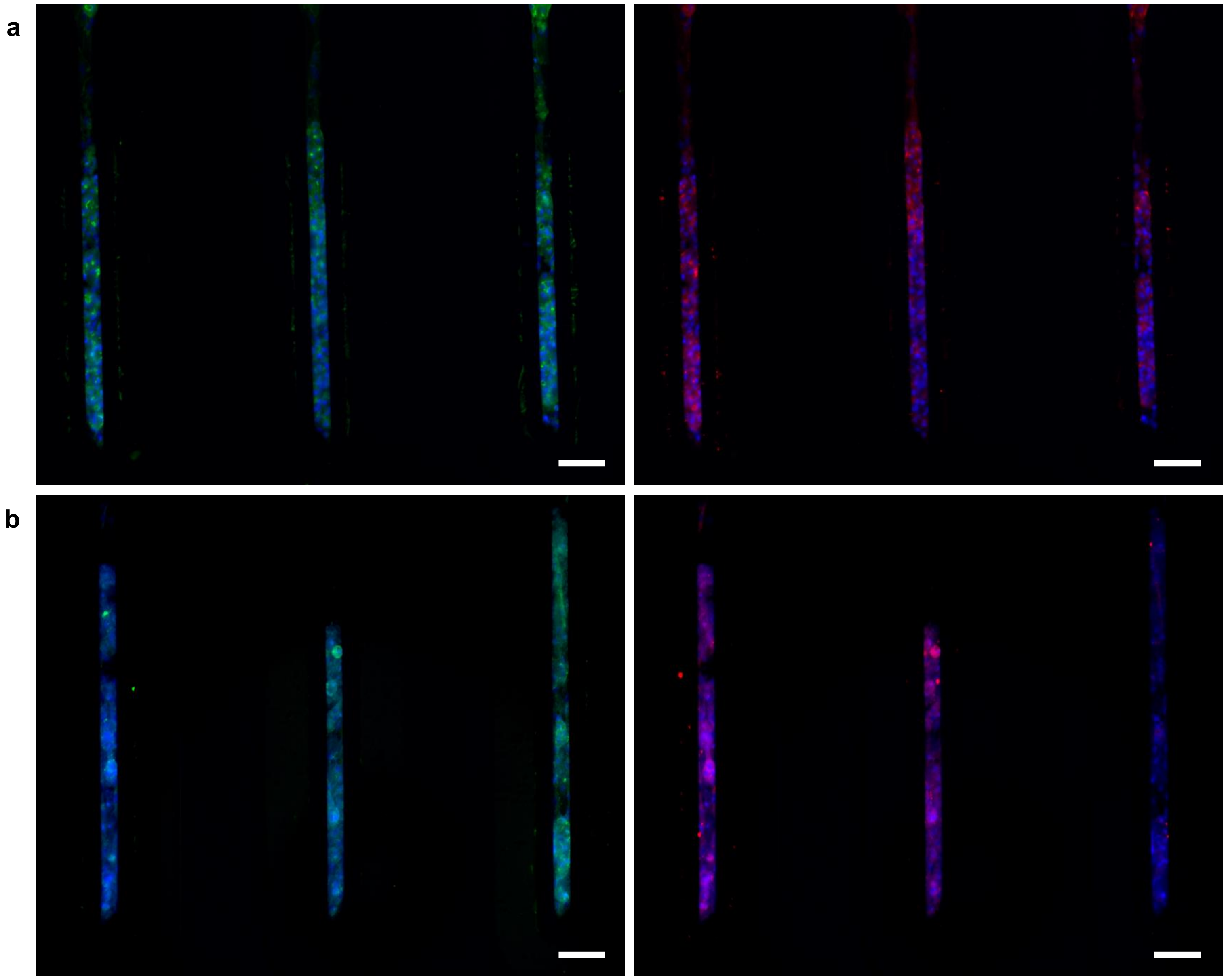

Detailed SU8 mould fabrication

Firstly, the design of the two levels of the microfluidic chip was transferred into chromium masks appropriate for use in UV photolithography. A two-level micro-mould made of thick photoresist (SU-8™, Kayaku Advanced Materials, Westborough, MA, USA) was then made using conventional clean room technologies. Details of the durations of the different steps are given in **Supplementary Table S2**. The first layer of the mould was 2 or 5 µm high (depending on the device), and defined the geometry of the slits connecting the hepatocyte chamber to the medium channel. SU-8 2002 or SU-8 2005 was spin-coated (3000 rpm, 300 rpm/s, 30s) on a silicon wafer to obtain an initial layer of 2 or 5 µm, respectively. The wafer was pre-baked at 65°C then 95°C and 65°C prior to UV exposure (365 nm, lamp power of 18 mJ/cm^2), then post-baked at the same temperatures and finally developed (SU-8 Developer, Kayaku Advanced Materials). SU-8 2025 was then spin-coated as a second layer (3000 rpm, 300 rpm/s, 30s for a 25 µm layer or 2000 rpm, 300 rpm/s, 30s for a 40 µm layer), and pre-baked prior to UV exposure and post-bake developing. Finally, the mould was hard baked in an oven (175°C, 2h). To make the PDMS chips, the mould was first incubated with hexamethyldisilazane vapours for 1h to achieve its passivation. PDMS monomer and reagent (SYLGARD 184 Silicone Elastomer, Dow Chemical, Midland, MI, USA) were mixed at 10:1 (v/v), degassed and poured onto the mould, which was baked for 2h at 75°C. The chip was then peeled off the mould. The four outlet holes were punched using a 1.5 mm puncher. To remove any remaining pieces of PDMS, the chip was sonicated in ethanol (96%) for 30 s and then dried. It was irreversibly bound to a glass coverslip (0.17 mm thick) with an oxygen plasma treatment (18 W, 300 mTorr, 20 s). Tubes about 1cm in length (teflon perfluoroalcoxy alcane (PFA), 0.50 mm internal diameter (ID), 1/16" outer diameter (OD)) were connected to each outlet, sealed using PDMS and baked for 1h at 75°C. Finally, the entire device was sterilized using oxygen plasma for 3 min.

Supplementary Table S2.

Layer height [µm]	SU-8 type	Pre-baking time (65°C; 95°C; 65°C) [min]	UV exposure time [s]	Post-baking time (65°C; 95°C; 65°C) [min]	Developing time [min]
2	2002	1; 1; 1	6	1; 2; 1	1
5	2005	1; 2; 1	14	1; 2; 1	2
25	2025	1; 7; 1	16	1; 10; 1	5
40	2025	1; 7; 1	20	1; 10; 1	5

Detailed immunostaining protocol

A negative pressure controller was used to perform immunostaining on chips because it generated a convective flow in the cell chambers and channels that is higher than in culture conditions, thanks to specific suction conditions. The syringe (and resistance if present) was disconnected from the inlet of the chip medium channel. The three outlets were connected to a pressure controller. The different solutions were deposited at the inlet and suction was started. Suction and static periods were then alternated as detailed in **Supplementary Table 3**.

Supplementary Table S3. Sequence of solutions being deposited and suctioned at one inlet of the chip to perform immunostaining on the device.

Solutions to be suctioned	Suction period [min]	Static period [min]
PBS washing	30	/
Fixation with 4% PFA	45	/
PBS washing	45	/
Permeabilisation with triton solution	45	20
PBS washing	30	/
Blocking with 3% BSA	45	60
Primary antibodies	60	/
**Overnight at 4°C**		
PBS with tween washing	45	/
Secondary antibodies and DAPI	45	120
PBS with tween washing	45	/
H_2O washing	45	/
**Chip imaging**		

Supplementary Table S4. Table of antibodies used during experiments.

	Antibody	Host and isotype	Company	Catalogue n°	Dilution
Primary antibodies	ALB	Mouse	Sigma	A6684	1/200
	HNF4α	Rabbit	Santa Cruz	SC-8987	1/200
	MDR3	Mouse	Chemicon	MAB4140	1/200
	ZO1	Rabbit	Novus Biologicals	NBP1-85047	1/200
Secondary antibodies	Alexa Fluor 488	Donkey anti-Mouse IgG	Fisher	A21202	1/1000
	Alexa Fluor 488	Donkey anti-Rabbit IgG	Fisher	A21206	1/1000
	Alexa Fluor 568	Donkey anti-Rabbit IgG	Fisher	A10042	1/1000
	Alexa Fluor 633	Donkey anti-Goat IgG	Fisher	A21082	1/1000
	Alexa Fluor 647	Donkey anti-Mouse IgG	Fisher	A31571	1/1000
	DAPI		Sigma	D9542	0.5 µg/ml
	Alexa Fluor 488 Phalloidin		Fisher	A12379	1/200
	Alexa Fluor 594 Phalloidin		Fisher	A12381	1/200

Supplementary Information References:

1. Buchwald, P. FEM-based oxygen consumption and cell viability models for avascular pancreatic islets. *Theor. Biol. Med. Model.* **6**, 1–13 (2009).
2. Curcio, E. *et al.* Mass transfer and metabolic reactions in hepatocyte spheroids cultured in rotating wall gas-permeable membrane system. *Biomaterials* **28**, 5487–5497 (2007).
3. Place, T. L., Domann, F. E. & Case, A. J. Limitations of oxygen delivery to cells in culture: An underappreciated problem in basic and translational research. *Free Radical Biology and Medicine* vol. 113 311–322 (2017).
4. Al-Ani, A. *et al.* Oxygenation in cell culture: Critical parameters for reproducibility are routinely not reported. *PLoS One* **13**, e0204269 (2018).
5. Evenou, F., Fujii, T. & Sakai, Y. Spontaneous formation of highly functional three-dimensional multilayer from human hepatoma hep G2 cells cultured on an oxygen-permeable polydimethylsiloxane membrane. *Tissue Eng. - Part C Methods* (2010)

doi:10.1089/ten.tec.2009.0042.
6. Markov, D. A., Lillie, E. M., Garbett, S. P. & McCawley, L. J. Variation in diffusion of gases through PDMS due to plasma surface treatment and storage conditions. *Biomed. Microdevices* **16**, 91–96 (2014).
7. Aleksandrova, A. V. *et al.* Mathematical and Experimental Model of Oxygen Diffusion for HepaRG Cell Spheroids. *Bull. Exp. Biol. Med.* (2016) doi:10.1007/s10517-016-3326-1.
8. Bavli, D. *et al.* Real-time monitoring of metabolic function in liver-onchip microdevices tracks the dynamics of Mitochondrial dysfunction. *Proc. Natl. Acad. Sci. U. S. A.* (2016) doi:10.1073/pnas.1522556113.
9. Nahmias, Y. *et al.* A novel formulation of oxygen-carrying matrix enhances liver-specific function of cultured hepatocytes. *FASEB J.* (2006) doi:10.1096/fj.06-6192fje.
10. Matsumoto, S. *et al.* Integration of an oxygen sensor into a polydymethylsiloxane hepatic culture device for two-dimensional gradient characterization. *Sensors Actuators, B Chem.* (2018) doi:10.1016/j.snb.2018.05.053.
11. Grünig, D., Felser, A., Bouitbir, J. & Krähenbühl, S. The catechol-O-methyltransferase inhibitors tolcapone and entacapone uncouple and inhibit the mitochondrial respiratory chain in HepaRG cells. *Toxicol. Vitr.* (2017) doi:10.1016/j.tiv.2017.05.013.
12. Peyta, L. *et al.* Reduced cardiolipin content decreases respiratory chain capacities and increases ATP synthesis yield in the human HepaRG cells. *Biochim. Biophys. Acta - Bioenerg.* (2016) doi:10.1016/j.bbabio.2016.01.002.
13. McCarty, W. J., Usta, O. B. & Yarmush, M. L. A Microfabricated Platform for Generating Physiologically-Relevant Hepatocyte Zonation. *Sci. Rep.* (2016) doi:10.1038/srep26868.
14. Waseem, N. & Chen, P. H. Hypoxic hepatitis: A review and clinical update. *Journal of Clinical and Translational Hepatology* (2016) doi:10.14218/JCTH.2016.00022.
15. Khakpour, S. *et al.* Oxygen transport in hollow fibre membrane bioreactors for hepatic 3D cell culture: A parametric study. *J. Memb. Sci.* (2017) doi:10.1016/j.memsci.2017.09.024.
16. Tanyeri, M., Ranka, M., Sittipolkul, N. & Schroeder, C. M. A microfluidic-based hydrodynamic trap: Design and implementation Flow in a rectangular channel. *Lab Chip* **11**, 1786–1794 (2011).
17. Schindelin, J. *et al.* Fiji: An open-source platform for biological-image analysis. *Nature Methods* (2012) doi:10.1038/nmeth.2019.
18. Čapek, M., Janáček, J. & Kubínová, L. Methods for compensation of the light attenuation with depth of images captured by a confocal microscope. *Microsc. Res. Tech.* (2006) doi:10.1002/jemt.20330.
19. Sage, D. *et al.* DeconvolutionLab2: An open-source software for deconvolution microscopy. *Methods* **115**, 28–41 (2017).
